# Reducing uncertainty in local temperature projections

**DOI:** 10.1126/sciadv.abo6872

**Published:** 2022-10-12

**Authors:** Saïd Qasmi, Aurélien Ribes

**Affiliations:** CNRM, Université de Toulouse, Météo-France, CNRS, Toulouse, France.

## Abstract

Planning for adaptation to climate change requires accurate climate projections. Recent studies have shown that the uncertainty in global mean surface temperature projections can be considerably reduced using historical observations. However, the transposition of these new results to the local scale is not yet available. Here, we adapt an innovative statistical method that combines the latest generation of climate model simulations, global observations, and local observations to reduce uncertainty in local temperature projections. By taking advantage of the tight links between local and global temperature, we can derive the local implications of global constraints. The model uncertainty is reduced by 30% up to 70% at any location worldwide, allowing to substantially improve the quantification of risks associated with future climate change. A rigorous evaluation of these results within a perfect model framework indicates a robust skill, leading to a high confidence in our constrained climate projections.

## Teaser

An innovative statistical method combines climate projections and observations to reduce uncertainty in local climate projections.

## INTRODUCTION

As the global mean temperature keeps rising and climate change intensifies, there is a growing demand for local-scale monitoring of current and future climate change. Assessing and planning the adaptation to the expected unprecedented impacts of climate change on human activities, ecosystems, and the biosphere as a whole require an accurate local information with well-calibrated uncertainties. This need relates to estimates of warming to date and the future warming in response to set of scenarios of future greenhouse gas emissions.

It is unequivocal that human influence has warmed the atmosphere, ocean, and land since preindustrial times (*1*). Concurrently, the anthropogenic influence is not detected everywhere at the local scale (*2*, *3*). Natural climate variability can blur the emergence of the anthropogenic signal for the next few years at high latitudes, while a substantial warming is already reported in several tropical regions (*4*, *5*). Regarding climate projections, the Intergovernmental Panel on Climate Change (IPCC) concluded in its fifth assessment report (AR5) (*6*) that “Future [human-induced] warming trends cannot be predicted precisely, especially at local scales”.

In the IPCC AR6 (*7*), a new generation of climate models (*8*) has been used to provide a range of projections in response to different socioeconomic scenarios (*9*). On the basis of this new dataset, various studies have recently shown that uncertainty in global mean warming can be considerably reduced using the information provided by recent observed warming trends via so-called “constraint” methods (*10*–*13*). These studies consistently point toward a downward revision of the expected warming in all emission scenarios (*10*, *12*), with a decrease in model uncertainty of nearly 40% for end of century projections (*11*), and even more at shorter lead times. This is an important result as, until then, observations have failed to provide clear evidence in reducing the range of climate projections (*14*).

The next challenge is to transpose these new findings on global warming to regional and local scales. At the regional scale, a few studies have adopted the partitioning from the Special Report on Managing the Risks of Extreme Events and Disasters to Advance Climate Change Adaptation (SREX) (*15*) and have attempted to narrow model uncertainty with sophisticated techniques with promising results (*16*, *17*). However, the SREX regions are typically continental-wide and do not provide relevant information for local adaptation. At the local scale (defined as the size of a global climate model grid box of about 200 km) and to the best of our knowledge, only a few studies have attempted to narrow climate model uncertainty, by using weighting methods to account for interdependencies between models (*18*, *19*) or by focusing on specific and limited areas (*20*–*22*). In particular, although constrained projections of global mean temperature are now used in the IPCC AR6 (*7*), local climate projections are still solely based on a raw ensemble of available climate models (https://interactive-atlas.ipcc.ch/), derived from global warming levels.

Here, we assess how much uncertainty in local temperature projections can be reduced. We first take advantage of the tight links that exist between local climate and global mean temperature (*23*, *24*). Specifically, we describe the local implications of the recent advances in the reduction of the uncertainties in global mean temperature projections. We then provide a set of local-scale temperature projections, which encapsulate another source of information: the observed local warming to date. If compared to the global mean temperature record, internal variability is larger in local observations. However, they still provide a useful source of information about both past and future trends, particularly over some specific regions. We discuss how much these two types of observations (global and local) narrow uncertainty on future warming ranges. Such a reduction is expected to provide more accurate information that becomes critical for policy-makers in the local climate risk management (*25*), as well as for the climate science community.

## RESULTS

The Kriging for Climate Change (KCC) method used by Ribes *et al.* (*11*, *16*) is one of the statistical techniques that have led to a significant reduction of uncertainty in probabilistic projections of global mean temperature by combining observations and models (*7*, *11*). This Bayesian method involves three steps. First, the response to all external forcings for each climate model considered is estimated over the period 1850–2100, after filtering out internal variability as much as possible. Second, the sample of forced responses from available climate models is used as a prior of the real-world forced response for each grid point. This is done assuming that “models are statistically indistinguishable from the truth.” Third, observations are used to derive a posterior distribution, i.e., a constrained temperature response, of the past and future forced response given observations, in a Bayesian way.

**Fig. 1.**
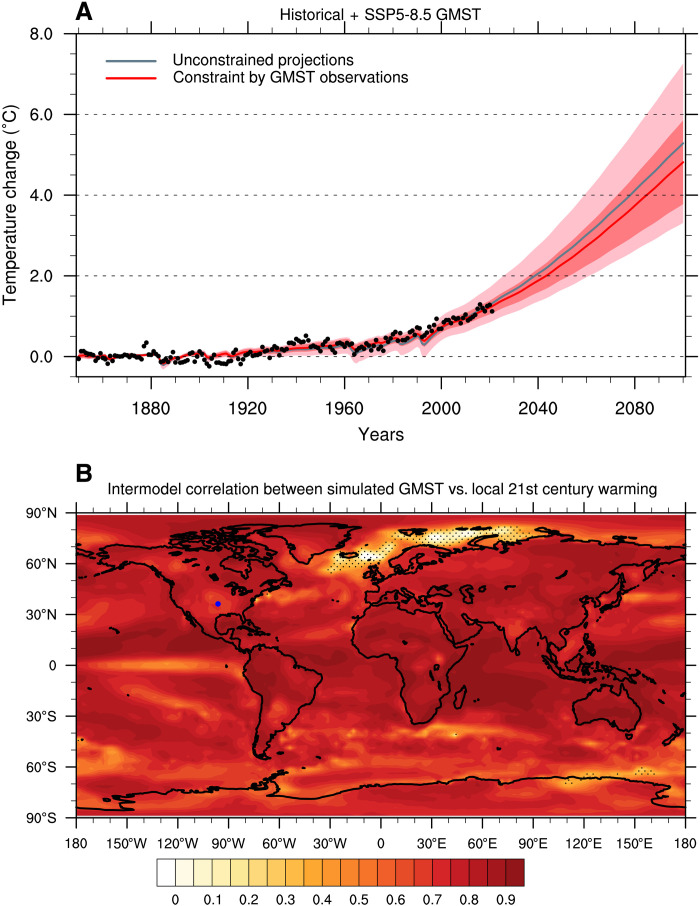
GMST time series and its correlation with local temperature. (**A**) GMST annual observations from the HadCRUT5 (*48*) dataset (black points) are used to constrain concatenated historical and SSP5-8.5 scenario simulations of GMST. The unconstrained (pink) and constrained (red) ranges stand for the 5 to 95% confidence interval of the forced response as estimated from 27 CMIP6 models. The thick pink (red) line stands for the ensemble mean (best estimate). All values are anomalies with respect to the 1850–1900 period. (**B**) Intermodel correlation between simulated GMST trends over the 2022–2100 period and local temperature trends over the 2022–2100 period. Stippling indicates regions with nonsignificant correlation (*P* > 0.05 based on a two-sided Student’s *t* test).

Compared to many emergent constraint approaches in which observations are often summarized into one single variable (*26*, *27*), the KCC method is able to take the full observed time series into account. Here, we further extend this technique to account for multiple time series and potential dependencies between them (see Materials and Methods). When applied to the global surface air temperature (GSAT) time series simulated by the models from the Coupled Models Intercomparison Project phase 6 (CMIP6) (*8*) models and the Shared Socioeconomic Pathway (SSP) 5 to 8.5 scenario, the amplitude of best estimate of the projected GSAT changes constrained by the observations is revised downward by 0.5°C by 2100, with a reduction in model uncertainty of nearly 40% (*11*). Here, we consider global mean surface temperature (GMST; a blending of land air and ocean sea surface temperatures; see Materials and Methods) instead of GSAT, as GMST is more consistent with local observational record. Figure 1A offers an update of the GMST constraint: the warming of 5.3°C projected by CMIP6 is in this case revised downward by 0.4°C (best estimate) in 2100. Minor differences with Ribes *et al*. (*11*) are explained by 2 years of additional observations, by the addition of several CMIP6 models, which affect the prior distribution, and by the lower warming observed in GMST compared to GSAT (*28*, *29*).

### Constrain local climate projections with global observations

**Fig. 2.**
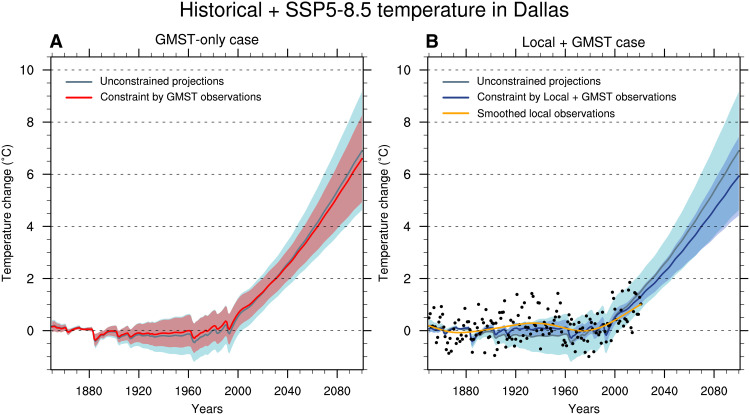
Observational constraints on historical and SSP5-8.5 local temperature changes in Dallas. (**A**) Constrained local temperature for the grid point at (48.75°N; 11.25°E) (see blue point in Fig. 1B) in the GMST-only case. The 5 to 95% constrained spread of the simulated response to all external forcings is in red, the thick line stands for the best estimate. The spread in light blue and the associated thick line is relative to the unconstrained case. (**B**) Constrained local temperature in the Local + GMST case. The 5 to 95% constrained spread of the simulated forced response is in blue, the thick line stands for the best estimate. Black points are the observations. Note that because single-year observations are affected by internal variability, they often lie outside the 5 to 95% assessed ranges of the forced responses. The orange line stands for the smoothed observations time series using 6° of freedom. Unconstrained simulated ranges are identical between (A) and (B). All values are anomalies with respect to the 1850–1900 period.

Climate models exhibit a strong correlation between future GMST and local warming over most regions of the globe (Fig. 1B). To take such a relationship into account, we extend the KCC method to constrain local temperature projections. Beyond the simple correlation shown in Fig. 1B, this method uses all the information contained in the entire observed GSAT time series to derive local warming (considering that the annual time series provides useful additional information, e.g., to distinguish between greenhouse gases (GHG) and aerosol forcings). This is done by deriving the local warming conditional on the observed GMST record (hereafter the GMST-only case; see Materials and Methods). As an example, we consider the North American city of Dallas for which the simulated local temperature over the 2022–2100 period is significantly correlated with future GMST (see the corresponding point in Fig. 1B). Consistent with GMST results, the local temperature range constrained by GMST observations indicates a decrease in uncertainty of about 20% over the 2021–2040 period, up to 30% over the 2081–2100 period (Fig. 2A) in the GMST-only case. The best estimate of local warming is revised downward by 0.4°C by 2100 compared to the unconstrained projections (hereafter the unconstrained case).

**Fig. 3.**
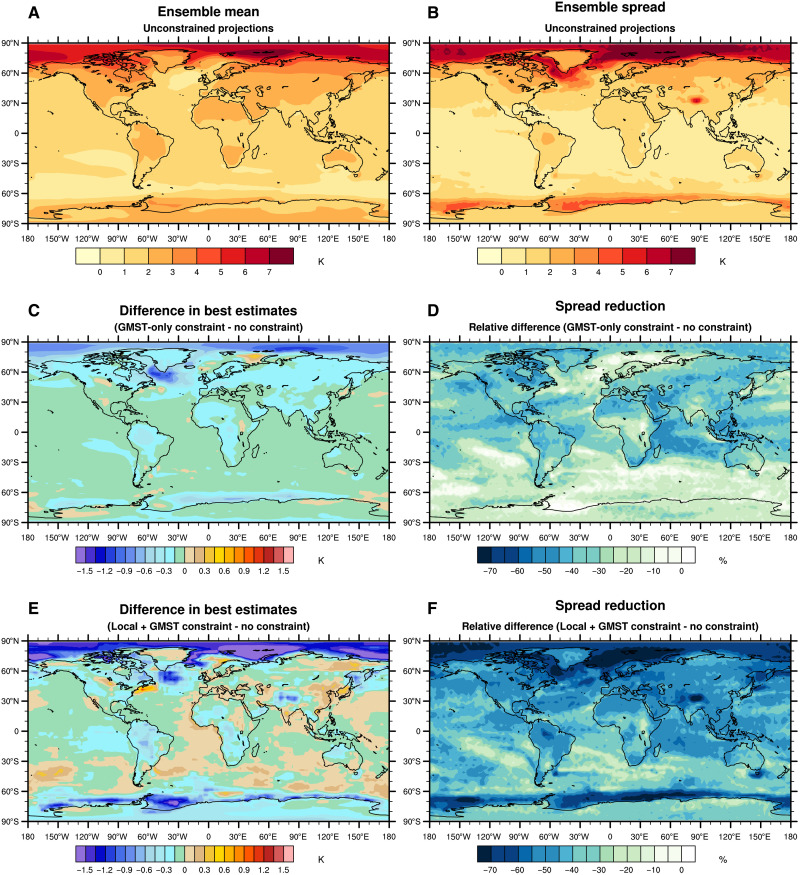
Unconstrained and constrained local temperature projections over the 2021–2040 period. (**A**) Ensemble mean of the unconstrained local temperature changes. (**B**) Ensemble spread of the unconstrained local temperature changes, defined as the 5 to 95% confidence interval of the multimodel ensemble. (**C**) Difference of local temperature changes ensemble mean between the GMST-only case and the unconstrained case. (**D**) Relative difference of local temperature changes ensemble spread between the GMST-only case and the unconstrained case. (**E**) Same as (C) but for the Local + GMST case. (**F**) Relative difference of local temperature changes ensemble spread between the Local + GMST case and the GMST-only case, illustrating how much incorporating local observations narrows uncertainty. All values are anomalies with respect to the 1850–1900 period.

**Fig. 4.**
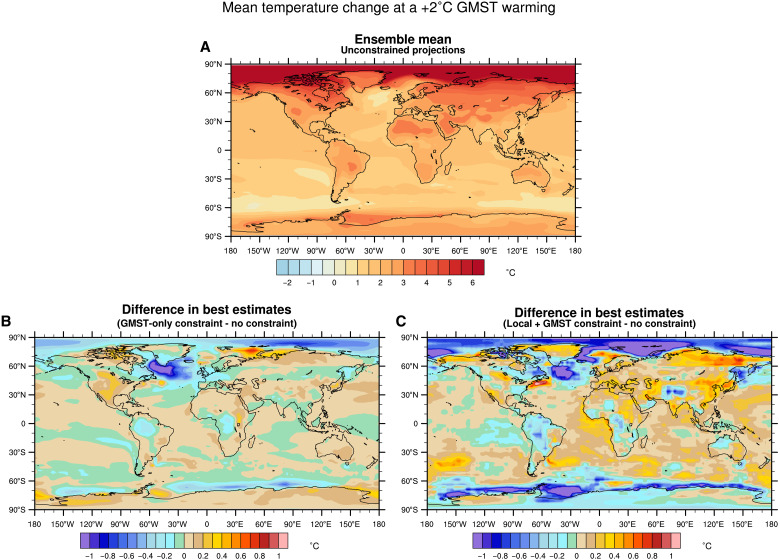
Warming pattern at +2°C of GMST warming. (**A**) Ensemble mean of the unconstrained local temperature changes. (**B**) Difference of local temperature changes between the GMST-only case (best estimate) and the unconstrained case (ensemble mean). (**C**) Difference of local temperature changes between the Local + GMST case (best estimate) and the unconstrained case (ensemble mean). By construction, global averaged differences are zero, as the GMST threshold remains at +2°C with or without constraint (contrary to Fig. 3). All values in (A) to (C) are anomalies with respect to the 1850–1900 period.

When the method is applied to any location worldwide, the results in the projected mean temperature and in model uncertainty show a clear relationship with the level of correlation between the local temperature and GMST (Figs. 3, B and D, and 1B). Note that the method is applied for each location separately, i.e., that it does not take advantage of spatial correlations and does not provide constrained projection at a larger regional scale. The reduction of uncertainty in local projections is the highest at the locations where the correlation with GMST is the strongest. For these locations, e.g., over several continental regions, the North Pacific and the Indian Ocean, a reduction of the ensemble spread of about 45% is obtained over the 2021–2040 period, with a downward revision of the best estimate warming between 0.2° and 1°C (Fig. 3, A and C). Conversely, for locations where the correlation is low, such as in southern Indian Ocean and the Barents Sea, the local temperature response is weakly constrained, with a reduction of the model uncertainty of 10% and a revision of the best estimate by 0.5°C or less. These revised ranges lead to a warming pattern at +2°C of global warming, considerably different from that assessed in the AR6 (Fig. 4, A and B, and fig. S1C). For example, the constrained local temperatures over North America are expected to be 0.4°C warmer compared to the unconstrained case. Note that, by construction, only the spatial pattern of warming is affected by the observational constraint in this case, since the +2°C warming level is let unchanged—so, the map of differences necessarily mixes patches of positive and negative values here, even if warming ranges at a given date are all revised downward (see caption of Fig. 4).

### Added value of local observations to the constraints

Beyond the useful information provided by the historical GMST time series, it is insightful to assess the consistency between the expected local response (regardless whether observed GMST is accounted for) and local historical observations. Current and past warming is spatially heterogeneous, and some regions such as the Arctic are warming faster than others (Fig. 3A) (*30*). Therefore, it is relevant to account for both GMST and local observations to provide local projections consistent with all available observations. Using recent local observations could particularly affect short-term projections (typically over the 2021–2040 period) and could provide a different picture of the constrained temperature ranges.

To make such a calculation, we derive a posterior of the expected local warming given local historical observations in addition to the GMST observations (hereafter the Local + GMST case; see Materials and Methods). Following the example of Dallas considered in Fig. 2A, the constrained local temperature ranges become typically closer to long-term local observed variations (Fig. 2B compared to Fig. 2A). The constraint by local observations is found to influence the best estimate of future warming even on longer lead times. Regarding the uncertainty, the added value of local observations in the reduction of the model spread compared to the GMST-only case is limited in this example, with a decrease of about 10% of the confidence range width compared to the GMST-only case. Two reasons contribute to this limited impact and must be considered for any location. First, the local signal-to-noise ratio can be small. This may happen if local internal variability or measurement uncertainty is large (i.e., local observations provide little insight on the externally forced response). Second, the global and local responses can be highly correlated with each other so that they partly provide the same information, leading to a limited impact of local observations on uncertainty ranges. In both cases, the model uncertainty will be only marginally reduced by local historical data (see Materials and Methods).

The application of the Local + GMST constraint to all grid points worldwide results in a projected warming pattern, which remains quite close to the GMST-only case (Fig. 3, C and E) but with regional differences. On the one hand, for several regions over the Arctic, the warming is revised downward compared to the unconstrained case, making the projections more consistent with recent observations and implying a reduced warming compared to the GMST-only case. On the other hand, an upward revision is obtained over Eastern Asia, Greenland, the East Siberian region, and Southern oceans. The added value of local observations in the reduction of model uncertainty is largest over these regions where the correlation in Fig. 1 is low (Fig. 3F). The estimated projections for the 2081–2100 period can also be derived and are consistent with these results (fig. S2). Note that even if each grid point is treated independently from the others (see Materials and Methods), the global mean of the constrained local ranges (for both the GMST-only and the Local + GMST cases) is very close to the constrained GMST ranges shown in Fig. 1A, suggesting a consistency between the different types of constraints.

The addition of local information can clearly modify the warming pattern at +2°C of global warming (Fig. 4, B and C; and fig. S1, B and C). For example, while a downward revision of the temperature change of −0.2°C is obtained over Europe in the GMST-only case, an upward revision of 0.3°C is obtained in the Local + GMST case. This change of sign is widespread over Eurasia. In the context of an urgent need of adaptation to the threat of climate change, our constrained warming pattern provides a revised and a more relevant information for local adaptation planning.

### Evaluation of the constrained projections

The robustness of these promising results is quantified within a so-called perfect model framework, using a leave-one-out cross validation (see Materials and Methods). Each member of each model is considered as pseudo-observations over the 1850–2021 period. These are subsequently used to constrain the temperature projections, using all other models as a prior. The constrained temperature range is then compared to the projected warming simulated by the model from which pseudo-observations were taken. As making this evaluation for all the grid points is computationally expensive, this procedure is applied to several locations, considered as representative of the diversity of the worldwide climate. As for the real observations, we assess both the GMST-only and Local + GMST constraints. The continuous ranked probability skill score (CRPSS) (*31*) is used to measure the accuracy of the method, taking the unconstrained projections as a baseline as a first step (see Materials and Methods).

**Fig. 5.**
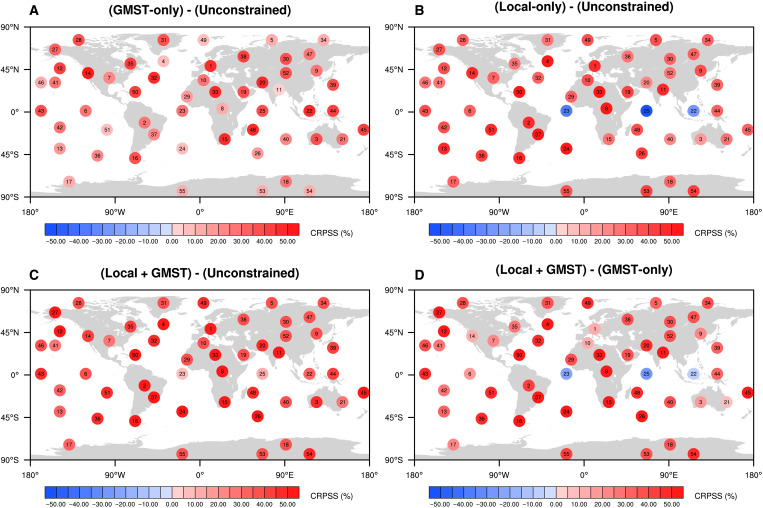
Spatial distribution of the CRPSS for the constrained temperature projections. The values indicate the average values from the CRPSS distributions (based on all pseudo-observations; see Materials and Methods) for the GMST-only (**A**), Local-only (B), and Local + GMST (**C**) constraints, respectively, compared to the unconstrained case for 55 locations (number in black over each point). (**D**) The added value of the local observations and stands for the CRPSS average in the Local + GMST case compared to the GMST-only case. For each location, the average is weighted by the number of members in each model. Calculations are made over the 2021–2040 period.

Figure 5A shows that the average of the CRPSS distribution based on all pseudo-observations is positive for every location in the GMST-only case with an improvement of about 30% over the 2021–2040 period. Depending on the location, the skill is remarkably improved by 10 to 50% (for the average) (Fig. 5 and figs. S5 to S9). In the Local + GMST case, the average skill is also positive (Fig. 5C), and for most locations, the CRPSS lies between 10 and 60% relative to the unconstrained case.

These results clearly demonstrate the performance of the method over short lead times (2021–2040). Moreover, the comparison between the Local + GMST and the GMST-only constraints indicates that the skill is slightly improved when adding local observations to constrain projections. The average of the CRPSS distribution is positive for 52 locations over 55 when comparing the Local + GMST and the GMST-only constraints (Fig. 5D and see boxplots in magenta in figs. S5 to S9) and is slightly negative for the remaining points. The significance of this result is assessed with a binomial test. Under the null hypothesis that adding local observations has no impact on the skill (i.e., that the GMST-only and Local + GMST cases are categories equally likely, such as a coin toss), the probability of getting this result by pure chance is less than 0.1%. This suggests that there is a clear added value in considering the constrained ranges derived from the Local + GMST case relative to the GMST-only case. A third case for which we only use local observations (Local-only case) to constrain projections indicates lower scores than in the Local + GMST case (Fig. 5B) and confirms that using the combination of global and local observations enhances the accuracy of the method. Similar results are obtained when we consider the 2081–2100 period (fig. S11).

The rare cases where the CRPSS is negative are due to the large low-frequency variability in few CMIP6 models. This is a topic of concern, as several CMIP6 model are characterized by clear multidecadal and even centennial internal variability in GMST (*32*, *33*). Figures S12 to S16 show that models associated with a strong decadal variability (e.g., CNRM-CM6-1, EC-Earth3, and IPSL-CM6A-LR) are those contributing negatively to the CRPSS in most cases. The assumptions used in the KCC method to statistically model internal variability might explain the failure to capture such a slow internal variability, resulting in overconfidence. Excluding those specific models from the evaluation process leads to a clear increase in the CRPSS by 15% both in the GMST-only and Local + GMST cases (fig. S17). Discussing the realism of these particular models requires further analyses on internal variability (*34*, *35*) and climate sensitivity (*36*), which are beyond the scope of this study. Still, regardless of whether the real system contains such low frequency variability, the CRPSS distributions remain mostly positive across locations for the three types of constraint, even if models with large internal variability are included. Both distributions in fig. S17 (excluding models with large internal variability) are above the distributions (including all models) in fig. S10, which themselves show mostly positive values (see the median value). Last, the relevance and reliability of our method seems robust to including all CMIP6 models and would be even strengthened if low-variability models were proven less realistic.

This is supported by a second evaluation criterion of the method based on coverage probabilities, which lead to similar conclusions (see Supplementary Discussion). From all of these evaluation results, we retain the Local + GMST case to provide guidance in constraining local projections. The evaluation of the KCC method suggests that the constrained temperature ranges are reliable and demonstrate that relying on unconstrained projections to assess the local future climate is no longer the best approach.

## DISCUSSION

**Fig. 6.**
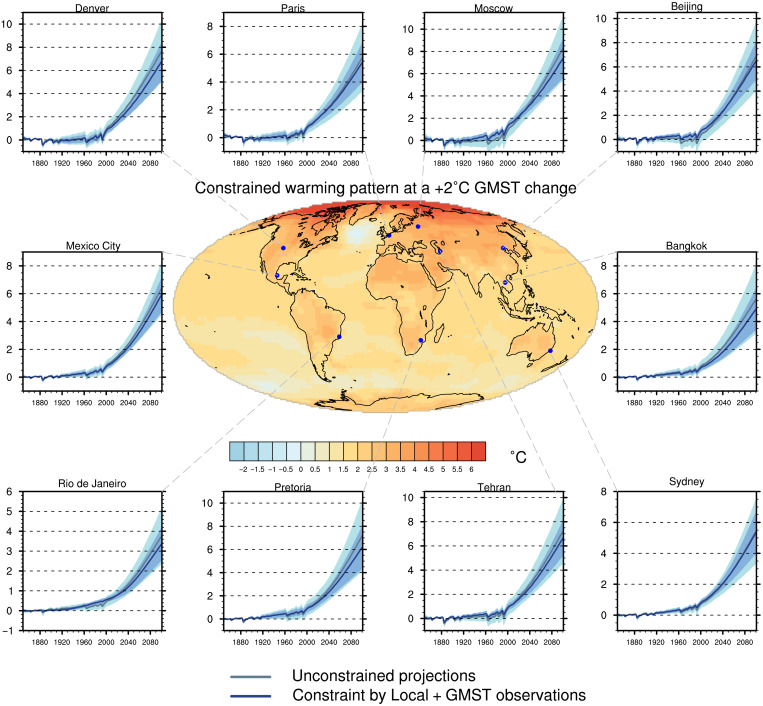
Mean temperature change at a +2°C GMST warming. Best estimate of the constrained local temperature changes in the Local + GMST case. Similarly to Fig. 2B, the constrained and unconstrained temperature ranges are shown for several world capitals cities over the 1850–2100 period. All values are anomalies with respect to the 1850–1900 period.

We have shown, using a statistical method combining the entire temperature observation records with model simulations, that uncertainty in local temperature projections can be substantially narrowed. Local projections constrained by both global and local observations exhibit a reduction of the uncertainty of 40% on average by 2100. This demonstrates the benefits of merging model simulations with observations to provide the best picture of future climate change. Figure 6 offers a complementary perspective to the AR6 (*7*) conclusions that were solely based on raw (unconstrained) projections. For each location, a temporal evolution from 1850 to 2100 of the constrained temperature and its uncertainty can be derived, with revised projections for the near and the long-term time scales. In particular, the KCC method provides a way out of the concept of global warming levels, by estimating the uncertainty for a given date. This fills the gap in the IPCC atlas (only based on unconstrained projections) and provides a considerable revision of the local exposure to the consequences of the on-going climate change (*37*). An online tool that implements the method and illustrates the constrained temperature ranges for every point over a horizontal grid of 2.5° resolution is available via the following demonstrator: https://saidqasmi.shinyapps.io/KCC-shinyapp/.

Promising prospects exist to improve the constrained projections. CMIP5 (*38*) and CMIP6 (*8*) ensembles sample model uncertainty in a probabilistic way using all climate models as an “ensemble of opportunities” (*39*, *40*). This approach has several limitations that can bias the estimation of climate uncertainty (*41*). First, they are not designed to sample uncertainty comprehensively (*42*), e.g., no models are being intentionally built to have low or high climate sensitivities. Forcing uncertainty is also poorly sampled in the CMIP6 ensemble, e.g., the magnitude of the aerosol forcing in 2014 (*43*). Second, each model output is considered as independent and contributes equally to the multimodel ensemble, although it is known that many CMIP6 models share common components and parametrizations (*44*). This “model democracy” paradigm has been largely used to summarize projection information in IPCC assessment reports (*6*), although it can be criticized (*45*). Therefore, using a subset of models qualified as independent a priori or weighting the models in this way (*46*, *47*), before applying the observational constraint, may provide even more reliable results.

Our results demonstrate that available observations offer valuable information to reduce uncertainty in climate projections over the next decades. Even with the continuous improvement of climate models, the intermodel spread is not necessarily reduced for several variables from one CMIP generation to the next (*1*). In this sense, the contribution of observations and constraint methods is expected to improve the reliability of the projections. Therefore, it is critical to account for this new source of information and to regularly bridge the gap between monitoring recent changes and predicting future changes.

The KCC method itself can also be improved. Although it can be used on larger areas to easily derive constrained projections, e.g., on the SREX regions (*16*), the current implementation does not take into account the spatial dependence in the climate variability between locations. Taking the spatial dimension fully into account could bring additional useful information and would result in consistent uncertainties at all spatial scales. This requires dealing with much larger covariance matrices. Estimating those matrices is a key challenge, as the number of models is much smaller than the number of grid points and years. Reducing the dimension of the problem, with methods used in data assimilation or considering spatiotemporal principal components may help to regularize these high-dimensional matrices.

Generalizing the method to other variables of high societal impacts, e.g., extreme precipitation, droughts, and snow cover, some of which are also tightly related to GMST, would also be very relevant. For some of these variables, the observed data are sparse, which requires finding well-sampled covariables over the historical period. In addition, variables such as sea ice or precipitation do not necessarily follow Gaussian distributions, which makes it necessary to adapt the KCC method to other types of distributions. In this way, the climate science community could take a step forward toward a more accurate assessment of past and future human-induced climate change.

## MATERIALS AND METHODS

### Observational dataset and models

The temperature observations are from the HadCRUT5 (*48*) dataset over the 1850–2021 period. The temperature field comes from a blending of near-surface temperature and sea surface temperature using land sea mask and sea ice concentration. The measurement uncertainty of the HadCRUT5 dataset is estimated from an ensemble of 200 equiprobable realizations. Most of the other observational products are included in the temperature range estimated by this ensemble, which confirms our choice to consider the HadCRUT5 dataset as a reference.

CMIP6 models are selected according to the availability of the following data: at least 200 years of a preindustrial control (piControl) simulation; at least one member of a historical simulation and one member of a projection simulation for the SSP5-8.5 scenario. To constrain the simulated temperatures at a grid point scale in a consistent way, a blended temperature field *T*_blend_ is computed in each CMIP6 model based on the formulation of Morice *et al.* (*48*)

$$\left\{ \begin{array}{c} w_{\text{air}}=(1-f_{\text{ocean}})+f_{\text{ocean}}f_{\text{ice}}\\ T_{\text{blend}}=w_{\text{air}}T_{\text{air}}+(1-w_{\text{air}})T_{\text{ocean}} \end{array}\right.$$ (1)

where *T*_air_, *T*_ocean_, *f*_ice_, and *f*_ocean_ are for each grid point near-surface air temperature, sea surface temperature, sea ice concentration, and sea area fraction. The 27 models for which these variables are available and which satisfy the above criteria are listed in Table 1.

**Table 1.** List of the available CMIP6 models and the associated number of members in the historical and SSP5-8.5 simulations used to constrain temperature projections.

**Model simulation**	**Historical + SSP5-8.5**
ACCESS-CM2	1
ACCESS-ESM1-5	3
BCC-CSM2-MR	1
CanESM5-CanOE	3
CanESM5	50
CESM2	2
CESM2-WACCM	1
CNRM-CM6-1	6
CNRM-CM6-1-HR	1
CNRM-ESM2-1	5
EC-Earth3	7
EC-Earth3-Veg	3
FGOALS-g3	1
FIO-ESM-2-0	3
GFDL-ESM4	1
HadGEM3-GC31-LL	1
INM-CM4-8	1
INM-CM5-0	1
IPSL-CM6A-LR	6
MIROC6	3
MIROC-ES2L	1
MPI-ESM1-2-HR	2
MPI-ESM1-2-LR	10
MRI-ESM2-0	1
NESM3	1
NorESM2-MM	1
UKESM1-0-LL	5
27 models	121 members

We define the GSAT as the global mean of *T*_air_ and the GMST as the global mean of *T*_blend_. Several studies have shown that GMST and GSAT clearly differ as GMST warms less than GSAT (*28*, *29*).

Models are interpolated on a common horizontal grid of 2.5° resolution before calculating blended temperatures and applying the constraining method. This choice is motivated by a compromise between the different resolutions of the CMIP6 models (between 1.5° and 2.5°). Note that the KCC method can be applied to finer resolutions if observations are available at this scale. For temperature, for which the spatial autocorrelation is high, the reduction in the uncertainty is expected to be the similar as for the 2.5° resolution.

### Statistical method

The statistical method is based on the same one used by Ribes *et al.* (*11*), whose formulation and principle is similar to kriging, which is a method originally developed to interpolate geophysical data based on prior covariances. In Ribes *et al.* (*11*), this method is applied to the analysis of time series from climate simulations of CMIP5 and CMIP6 models and is used for several purposes: (i) reducing model uncertainty on past and future global warming estimated by CMIP and ScenarioMIP (*9*) simulations, (ii) reducing uncertainty on warming attributed to several external forcings via the Detection and Attribution Model Intercomparison Project (DAMIP) (*49*) models, and (iii) complete or statistically reconstruct missing simulations from other physically relevant simulations (e.g., using the so-called 1% CO_2_ simulations in which the CO_2_ concentration increases by 1% each year, to reconstruct DAMIP historical simulations in which greenhouse gases follow their historical concentrations, while other forcings are kept constant). Here, we apply this method of KCC to reduce the model uncertainty in the past and future temperature changes simulated by CMIP6 models at each grid point. Note that a confusion can be made with techniques based on so-called emergent constraints methods (*26*, *27*). Emergent constraints would usually consider the sole observed global warming trend (a single scalar); e.g., over the 1980–2021 period, to constrain the simulated temperature changes in the future. The KCC method has several advantages compared to this approach. Instead of simply constraining a trend over a subperiod, it uses the entire observed time series of temperature, which avoids ignoring useful information. In addition, the method takes into account the model temporal pattern uncertainty and provides confidence ranges specifically for the forced response, while many other studies also include internal variability.

For a given grid point, we define $y_{\text{loc}}^{*}$ as the yearly time series of the real (and unknown) temperature response to external forcings over the 1850–2021 period and **y**_loc_ as the observed yearly temperature time series over the same period. Similarly, we define for the GMST the vectors $y_{\text{glo}}^{*}$ and **y**_glo_ as the unknown response to external forcings and the observed time series, respectively. They constitute the following y* and **y** vectors, both of size 2*n_y_* where *n_y_* = 170

$$y*=(\begin{array}{c} y_{\text{loc}}^{*}\\ y_{\text{glo}}^{*} \end{array}),y*=(\begin{array}{c} y_{\text{loc}}\\ y_{\text{glo}} \end{array})$$ (2)

Assuming that the observed temperature total variability can be decomposed as the sum of a term of forced variability and a term including both internal variability and measurement errors, **y** takes the following form

y=y*+ϵ (3)

where **ϵ** = (**ϵ**_loc_, **ϵ**_glo_) is a vector of size 2*n_y_* and corresponds to the local and global terms of measurement errors and observed internal variability. Further assuming that models are indistinguishable from the truth, i.e., that observations and models are exchangeable (*50*–*52*), observations **y** can be rewritten

$$\left\{ \begin{array}{c} x=(\begin{array}{c} x_{\text{loc}}\\ x_{\text{glo}} \end{array})\\ y=\mathrm{Hx}+\epsilon \end{array}\right.$$ (4)

where **x**_loc_ and **x**_glo_ are the yearly time series over the 1850–2100 period of the local and global temperature responses to external forcings estimated in CMIP6 models, respectively, i.e., vectors of size *n_x_* = 251. **H** is an observation operator of size 2*n_y_* × 2*n_x_*, which extracts the part of **x** that is observed in **y**, i.e., the forced response from 1850 to 2021, and whose form depends on the type of the applied constraint (using only GMST observations or both GMST and local observations; see eq. S21). Note that the assumption of exchangeability between observations and models has been suggested as well supported by observations, especially for temperature (*50*, *53*).

For a given CMIP6 model *m* listed in Table 1, we choose to estimate the simulated response to all external forcings **x**_*m*,glo_, by decomposing the simulated GMST over 1850–2100 into an anthropogenic response **x**_*m*,ant,glo_, and a natural response **x**_*m*,nat,glo_. Therefore, after averaging all available members of the model *m*, the simulated GMST time series over 1850–2100 **x**_*m*,glo_ writes

$$x_{m,\text{glo}}=x_{m,\text{nat},\text{glo}}+x_{m,\text{nat},\text{glo}}+\epsilon_{m}$$ (5)

where **ϵ***_m_* is a random term for internal variability.

To estimate **x**_*m*,nat,glo_ and **x**_*m*,ant,glo_ in the model *m*, we use a generalized additive model (GAM) to compute the response to all external forcings, **x**_*m*,glo_ (recall that **x**_*m*,glo_ follows Eq. 4)

$$\left\{ \begin{array}{c} x_{m,\text{glo}}=x_{m,\text{all},\text{glo}}+\epsilon_{m}\\ x_{m,\text{all},\text{glo}}=\underset{x_{m,\text{nat},\text{glo}}}{\underbrace{\beta_{m}e}}+\underset{x_{m,\text{ant},\text{glo}}}{\underbrace{f(t)}} \end{array}\right.$$ (6)

where β*_m_* is an unknown scaling factor. **e** is a vector of size *n_x_* and is the temperature response to the natural forcings computed from a two-layer (atmosphere-ocean) energy balance model (EBM) following equations 1 and 2 of Geoffroy *et al.* (*54*), using the historical and SSP5-8.5 natural forcings between 1850 and 2100 estimated by the Priestley Center (*55*) as a radiative term. Here, **e** is calculated using an average of EBM parameters fitted to the CMIP6 ensemble and aims at estimating rapid year-to-year variations of natural forcings. *f*(**t**) is a time series [with **t** = (1850, …,2100)] and refers to an assumed smoothed response of GMST to the anthropogenic forcings (i.e., a smoothed response of **x**_*m*,glo_). The function *f* corresponds to smoothing splines to filter out part of internal variability, with 6° of freedom (a value that was selected as a bias-variance trade-off). The total forced responses as estimated by this procedure are illustrated in fig. S18.

We apply the exact same procedure to estimate the local forced responses as simulated by each CMIP model. For each grid point from the model *m*, we consider **x**_*m*,loc_, the average of all available members, to estimate the local forced response, **x**_*m*,all,loc_. We assume that the local natural response scales linearly with the globally averaged natural forcings time series, as the EBM response **e** used to calculate **x**_*m*,nat,glo_ is also used when we fit the GAM to compute the local natural response **x**_*m*,nat,loc_. Thus, **x**_*m*,nat,glo_ and **x**_*m*,nat,loc_ only differ by their scaling factor β*_m_*. We believe that our results are not sensitive to this choice given the reduced strength of, and uncertainty in, the natural response compared to the anthropogenic response.

The multimodel ensemble of the local and global simulated responses to all external forcings is used to derive a distribution of **x**, noted Π(**x**) ∼ N(**μ**, Σ_mod_), built from all **x**_*m*,glo_ and **x**_*m*,loc_. **μ** = (**μ**_loc_, **μ**_glo_) is a vector of size 2*n_x_* and is the multimodel ensemble mean of the concatenated local and global forced responses. Σ_mod_ is a variance-covariance matrix of size 2*n_x_* × 2*n_x_* that describes the model spread, with the following form

$$\Sigma_{\text{mod}}=[\begin{array}{cc} \Sigma_{\text{mod},\text{loc}} & \Sigma_{\text{mod},\text{dep}}\\ \Sigma_{\text{mod},\text{dep}}^{\prime } & \Sigma_{\text{mod},\text{glo}} \end{array}]$$ (7)

where Σ_mod,loc_ and Σ_mod,glo_ are the sample covariance matrices of size *n_x_* × *n_x_* modeling local and global model spread within **x**_loc_ and **x**_glo_, respectively. Σ_mod,dep_ is the covariance matrix modeling the dependence between **x**_loc_ and **x**_glo_

In our Bayesian framework, Π(**x**) is a first (probabilistic) estimate of **x**, which makes no use of observations, and is only based on climate models. We want to update this estimate by incorporating the observational evidence provided by **y**. Following the Bayesian theory, the calculation of the posterior distribution *p*(**x**∣**y**) is required. A prerequisite is to define the observational uncertainty, i.e., the covariance matrix associated with **y**.

### Modeling of observational uncertainty

Given Eq. 4, we assume that **ϵ** ∼ N(**0**, Σ_obs_), where Σ_obs_ = Σ_meas._ + Σ_iv_ is the observation error covariance matrix. Σ_meas._ and Σ_iv_ are both of size 2*n_y_* × 2*n_y_* and describe the measurement error and internal variability, respectively. Σ_meas._ is estimated as the sample covariance matrix over the 200-member ensemble of the HadCRUT5 dataset (*48*).

Σ_iv_ is estimated using observed annual time series of global and local temperature over the 1850–2021 period. First, we compute the global observational residuals by subtracting the CMIP6 response to all external forcings **μ**_glo_(1,…,*n_y_*) from the observations **y**_glo_. Similarly, we derive local residuals by subtracting **μ**_loc_(1,…,*n_y_*) from **y**_loc_. These residuals constitute a first estimate of global and local internal variability, noted ${\hat{\epsilon }}_{\text{iv},\text{loc},1},{\hat{\epsilon }}_{\text{iv},\text{glo},1}$, respectively.

We define Σ_iv_ as a matrix of size 2*n_y_* × 2*n_y_* of the following form

$$\sum_{\text{iv}}=[\begin{array}{cc} \Sigma_{\text{iv},\text{loc}} & \Sigma_{\text{iv},\text{dep}}\\ \Sigma_{\text{iv},\text{dep}}^{\prime } & \Sigma_{\text{iv},\text{glo}} \end{array}]$$ (8)

where Σ_iv,loc_ and Σ_iv,glo_ are the covariance matrices of size *n_y_* × *n_y_* modeling local and global internal variability within **y**_loc_ and **y**_glo_, respectively. Σ_iv, dep_ is the covariance matrix modeling the dependence between local and global internal variability, i.e., **ϵ**_iv,loc_ and **ϵ**_iv,glo_.

To compute Σ_iv_, we take into account decadal internal variability that exists in the global (*56*), regional (*57*), and even local (*58*) observations, using a mixture of two autoregressive processes or order 1 (AR1), hereafter mixture of autoregressive processes (MAR), as done by Ribes *et al.* (*11*). The MAR formulation includes a fast (f) and a slow (s) components such that global internal variability **ϵ**_iv,glo_ within the GMST residuals writes at a time *t*

$$\left\{ \begin{array}{c} \epsilon_{\text{iv},\text{glo}}(t)=\epsilon_{\text{iv},f,\text{glo}}(t)+\epsilon_{\text{iv},s,\text{glo}}(t),\\ \epsilon_{\text{iv},f,\text{glo}}(t)=\alpha_{f,\text{glo}}\epsilon_{\text{iv},f,\text{glo}}(t-1)+Z_{f,\text{glo}}(t),\\ \epsilon_{\text{iv},s,\text{glo}}(t)=\alpha_{s,\text{glo}}\epsilon_{\text{iv},s,\text{glo}}(t-1)+Z_{s,\text{glo}}(t) \end{array}\right.$$ (9)

where the parameters α_s,glo_ and α_f,glo_ are the lag 1 coefficients of the AR1 processes and α_s,glo_ ≥ α_f,glo_ by convention. $Z_{s,\text{glo}}(t)∼N(0,\sigma_{s,\text{glo}}^{2})$ and $Z_{f,\text{glo}}(t)∼N(0,\sigma_{f,\text{glo}}^{2})$ are white noises associated with the two AR1. The slow component is able to generate a dependence on time scales of typically one decade, while the fast component accounts for interannual variability. Following the principle of parsimony, only four coefficients $\left(\sigma_{f,\text{glo}}^{2},\alpha_{f,\text{glo}},\sigma_{s,\text{glo}}^{2},\text{and}\;\alpha_{s,\text{glo}}\right)$ are thus needed to characterize internal variability at the global scale and to make Σ_iv,glo_ invertible. We fill the covariance matrix Σ_iv,glo_ following the calculations of each of its coefficients, as detailed in eq. S8. In practice, we apply a maximum likelihood procedure to the local and global residuals according to the statistical model from Eq. 9. Uncertainty related to these coefficients is not taken into account. Then, we make the same assumptions, and estimate four other parameters, $\left(\sigma_{f,\text{loc}}^{2},\alpha_{f,\text{loc}},\sigma_{s,\text{loc}}^{2},\text{and}\;\alpha_{s,\text{loc}}\right)$, to characterize fast and slow components in local internal variability **ϵ**_iv, loc_ and to compute Σ_iv, loc_.

The initial estimate of Σ_iv_, noted ${\hat{\Sigma }}_{\text{iv},1}$ is solely based on the residuals ${\hat{\epsilon }}_{\text{iv},\text{loc},1}$ and ${\hat{\epsilon }}_{\text{iv},\text{glo},1}$ derived from the unconstrained forced response. This first estimate is likely flawed as the real (and unknown) forced response y* is not necessarily consistent with the unconstrained forced response estimated by **μ**. In addition, as **μ** can be by construction different from the best estimate of the constrained forced response $\hat{\mu_{1}}$ (the mean of the posterior distribution *p*(**x**∣**y**), see section bellow), the residuals ${\hat{\epsilon }}_{\text{iv},\text{loc},1},{\hat{\epsilon }}_{\text{iv},\text{glo},1}$ before constraint are not always coherent with the residuals ${\hat{\epsilon }}_{\text{iv},\text{loc},2},{\hat{\epsilon }}_{\text{iv},\text{glo},2}$ computed as the $y-\hat{\mu_{1}}$ difference.

Hence, to ensure an accurate estimation of internal variability in the constraint procedure, an iterative algorithm is applied to find the MAR parameters that fit the residuals from the constrained forced response

$$\begin{array}{c} y-\mu \overset{\text{residuals}}{\to }{\hat{\epsilon }}_{\text{iv},1}\overset{\text{constraint}}{\to }{\hat{\mu }}_{1}\\ y-{\hat{\mu }}_{1}\overset{\text{residuals}}{\to }{\hat{\epsilon }}_{\text{iv},2}\overset{\text{constraint}}{\to }{\hat{\mu }}_{2}\\ y-{\hat{\mu }}_{n-1}\overset{\text{residuals}}{\to }{\hat{\epsilon }}_{\text{iv},n}\overset{\text{constraint}}{\to }{\hat{\mu }}_{n} \end{array}$$ (10)

where, for each iteration *n*, $\hat{\mu_{n}}$ and ${\hat{\epsilon }}_{\text{iv},n}$ are estimates of the forced response and internal variability, respectively. The termination criterion is based on the Frobenius norm ‖. ‖*_F_*. Hence, we consider that the algorithm converges at the iteration *n*, i.e., that ${\hat{\epsilon }}_{\text{iv},n}\to \epsilon_{\text{iv}}$, when the relative difference between ${\left\|{\hat{\Sigma }}_{\text{iv},n}\right\|}_{F}$ and ${\left\|{\hat{\Sigma }}_{\text{iv},n-1}\right\|}_{F}$ is inferior to 1%, meaning that the MAR parameters values have also converged. In practice, *n* varies between 2 and 4 depending on the location.

**Fig. 7.**
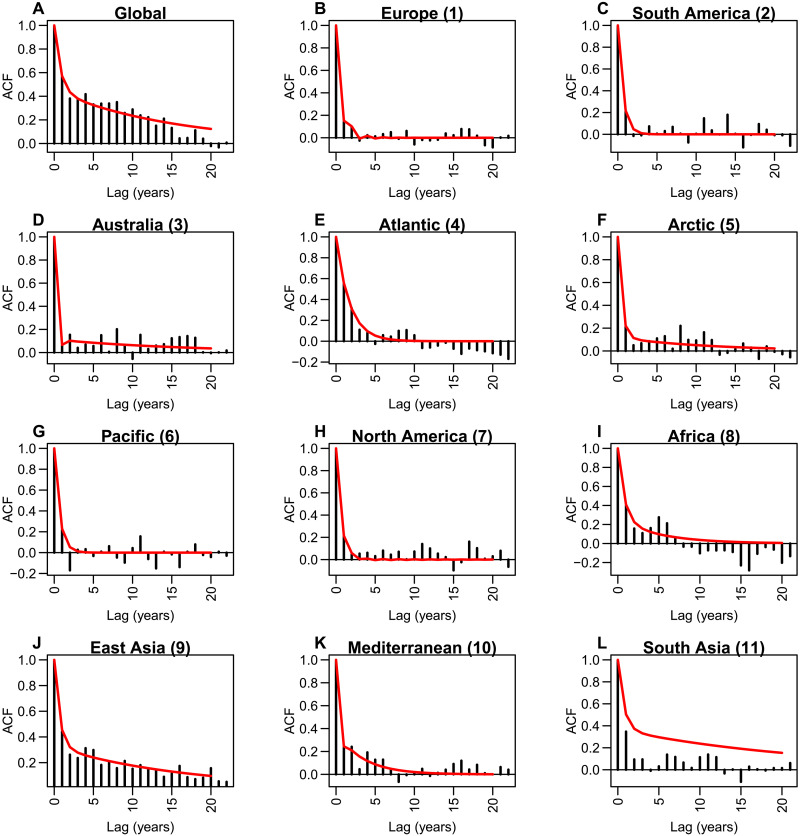
Estimation of observed internal variability. (**A**) Autocorrelation function (ACF) of the GMST observations (bars) after applying the iterative algorithm (see Materials and Methods) to the CMIP6 multimodel mean forced response and the GMST observed time series. The ACF of the MAR processes fitted to the same residual time series according to Eq. 9 is in red. (**B** to **L**) Same as (A) but for local residuals from 11 selected points (see Fig. 5).

The autocorrelations from this MAR model suggest that our statistical representation of internal variability effectively captures decadal variability (typically between lag 5 and lag 10) in the GMST and local temperature time series, e.g., for the Atlantic, African, and South American regions (Fig. 7). We are aware that initial condition large ensembles and long piControl simulations provide a nice sampling of internal variability and could also be used to estimate this variability. However, we choose to not directly rely on it because of the huge discrepancies between models in terms of their simulated internal variability (*56*). Figures S20 to S30 illustrate this aspect with the piControl simulations from the CMIP6 models, including those used to build large ensembles. In all cases, the models do not converge to a consistent estimate of internal variability. For instance, over the Atlantic ocean, many models exhibit clear pseudo-periodic low frequency variability, while other models do not simulate decadal variability.

### Modeling of the dependence between local and global internal variability

As impacts from Pacific and Atlantic decadal variability (and potential other modes of variability) on GMST have been reported over the historical period (*59*, *60*), we need to allow a potential dependence between global and local internal variability in Σ_iv,dep_. Therefore, finding a simple and parsimonious dependence model that is compatible with the MAR structure is required. Allowing the covariances Cov [ϵ_s,glo_(*t*), ϵ_s,loc_(*t*)] and Cov [ϵ_f,glo_(*t*), ϵ_f,loc_(*t*)] to be nonzero is not trivial, and these terms need to be quantified to fill the covariance matrix Σ_iv,dep_. Note that the fast and slow components remain always independent and that Σ_iv_ is computed for each location separately, as the spatial dependence among various locations is not considered in the method. To compute Σ_iv,dep_, we introduce a ninth parameter λ accounting for some correlation between the local versus global components in the MAR modeling. The formulation of the covariances is slightly different in this case, and the calculations are detailed in the Supplementary Materials.

### Calculation of *p*(x∣y)

As Π(**x**) and **ϵ** are assumed to follow normal distributions, the Gaussian conditioning theorem is applicable to derive the posterior or the “constrained” distribution *p*(**x**∣**y**). Its formulation (detailed in eq. S23) indicates that the method is conservative: The uncertainty in *p*(**x**∣**y**) is never larger than that in Π(**x**). Therefore, if observed internal variability is very large, then the model uncertainty in *p*(**x**∣**y**) will remain very close to that in Π(**x**).

### Perfect model evaluation

We evaluate the performance of the KCC method within a perfect model framework, following a leave-one-out cross-validation:

1) For a given model, we consider a single member as pseudo-observations **y** over the 1850–2021 period (the historical simulation is extended by the SSP5-8.5 simulation over the 2015–2021 period).

2) We use the other 26 models to derive the prior Π(**x**) ∼ N(μ, Σ_mod_).

3) As there is no measurement uncertainty in models, Σ_meas._ is null; therefore, Σ_obs_ = Σ_iv_. As done with the real observations, internal variability within the pseudo-observations is estimated from the difference between the pseudo-observations time series and the forced temperature response estimated by the ensemble mean of the 26 other models. Σ_iv_ is then derived from the MAR fitted on the obtained residuals.

4) We apply the KCC method using the inputs **y**, Σ_obs_, **μ**, Σ_mod_ to calculate projected changes constrained by pseudo-observations.

5) These four steps are repeated for each available member of the considered model and for all available models.

### Continuous ranked probability score

We use the CRPS (*31*) to quantify the performance of the KCC method. It is defined as the quadratic measure of discrepancy between (i) 1(*x* ≥ *y*_pobs_), the empirical cumulative distribution function (CDF) of a scalar pseudo-observation *y*_pobs_ simulated by one model and averaged over the future period, and (ii) the projected CDF *G*_cons_ of *p*(**x**∣**y**) (derived from all of the other models) over the same period

$${\text{CRPS}}_{\text{cons}}(G_{\text{cons}},y_{\text{pobs}})=\int_{\mathbb{R}}{\left[G_{\text{cons}}(x)-1(x\geq y_{\text{pobs}})\right]}^{2}\mathrm{dx}$$ (11)

where 1 is the indicator function (note that *x* is here a bound variable in the integral, different from the vector **x** in Eq. 4). Similarly, we define a reference CRPS, CRPS_ref_ based on *G*_ref_, the CDF of Π(**x**), the unconstrained distribution, and *y*_pobs_. We can compute the CRPSS, which quantifies the performance of the KCC method if compared to the reference

$$\text{CRPSS}=1-\frac{{\text{CRPS}}_{\text{cons}}}{{\text{CRPS}}_{\text{ref}}}$$ (12)

The CRPSS is computed over all available pseudo-observations (121 values; see Table 1). CRPS_cons_ is calculated in both GMST-only and Local + GMST cases. Therefore, the quantity $1-\frac{\text{CRP}S_{\text{cons}}(\text{Local}+\text{GMST})}{\text{CRP}S_{\text{cons}}(\text{Global}-\text{only})}$ allows quantifying the added value from local observations compared to the sole use of GMST observations. A positive (negative) value, indicates an improvement (deterioration). The higher the CRPSS (bounded at 1), the better the performance.

## Supplementary Materials

### This PDF file includes:

Supplementary Equations Supplementary Discussion Table S1 Figs. S1 to S31
