# Supplementary material for: Reducing uncertainty in local temperature projections
Source: Sci Adv. 2022 Oct 12;8(41):eabo6872. doi: 10.1126/sciadv.abo6872 (PMC9555774; doi:10.1126/sciadv.abo6872)
Supplement: Supplementary file 1 — Supplementary Equations Supplementary Discussion Table S1 Figs. S1 to S31 [file sciadv.abo6872_sm.pdf]

Science Advances
AAAS

Supplementary Materials for

Reducing uncertainty in local temperature projections

Saïd Qasmi and Aurélien Ribes

Corresponding author: Saïd Qasmi, said.qasmi@meteo.fr

Sci. Adv. **8**, eabo6872 (2022)
DOI: 10.1126/sciadv.abo6872

This PDF file includes:

Supplementary Equations
Supplementary Discussion
Table S1
Figs. S1 to S31

Supplementary Equations

The notations adopted in this section follow those of the Methods section in the main text.

Estimation of the covariance matrix $\mathbf{\Sigma}_{\mathrm{iv}}$

Recall that we define $\mathbf{\Sigma}_{\mathrm{iv}}$ as a matrix of size $2n_y \times 2n_y$ of the following form:

$$\mathbf{\Sigma}_{\mathrm{iv}} = \left[\begin{array}{c|c} \mathbf{\Sigma}_{\mathrm{iv,loc}} & \mathbf{\Sigma}_{\mathrm{iv,dep}} \\ \hline \mathbf{\Sigma}'_{\mathrm{iv,dep}} & \mathbf{\Sigma}_{\mathrm{iv,glo}} \end{array}\right], \tag{1}$$

where $\mathbf{\Sigma}_{\mathrm{iv,loc}}$ and $\mathbf{\Sigma}_{\mathrm{iv,glo}}$ are the covariance matrices of size $n_y \times n_y$ modeling local and global internal variability within $\boldsymbol{y}_{\mathrm{loc}}$ and $\boldsymbol{y}_{\mathrm{glo}}$, respectively. $\mathbf{\Sigma}_{\mathrm{iv,dep}}$ is the covariance matrix modeling the dependence between the two residuals.

Estimation of $\mathbf{\Sigma}_{\mathrm{iv,loc}}$ and $\mathbf{\Sigma}_{\mathrm{iv,glo}}$

First, following equation 9 in the main text, we decompose $\epsilon_{\mathrm{iv,f,glo}}(t)$ as an infinite sum using back substitutions:

$$\begin{aligned}
\epsilon_{\mathrm{iv,f,glo}}(t) &= \alpha_{\mathrm{f,glo}}\epsilon_{\mathrm{iv,f,glo}}(t-1) + Z_{\mathrm{f,glo}}(t), \\
&= \alpha_{\mathrm{f,glo}}[\alpha_{\mathrm{f,glo}}\epsilon_{\mathrm{iv,f,glo}}(t-2) + Z_{\mathrm{f,glo}}(t-1)] + Z_{\mathrm{f,glo}}(t), \\
&= \alpha^2_{\mathrm{f,glo}}\epsilon_{\mathrm{iv,f,glo}}(t-2) + \alpha_{\mathrm{f,glo}}Z_{\mathrm{f,glo}}(t-1) + Z_{\mathrm{f,glo}}(t), \\
&= \alpha^2_{\mathrm{f,glo}}[\alpha_{\mathrm{f,glo}}\epsilon_{\mathrm{iv,f,glo}}(t-3) + Z_{\mathrm{f,glo}}(t-2)] + \alpha_{\mathrm{f,glo}}Z_{\mathrm{f,glo}}(t-1) + Z_{\mathrm{f,glo}}(t), \\
&= \alpha^3_{\mathrm{f,glo}}\epsilon_{\mathrm{iv,f,glo}}(t-3) + \alpha^2_{\mathrm{f,glo}}Z_{\mathrm{f,glo}}(t-2) + \alpha_{\mathrm{f,glo}}Z_{\mathrm{f,glo}}(t-1) + Z_{\mathrm{f,glo}}(t), \\
&= \vdots \\
&= \sum_{k=0}^{\infty} \alpha^k_{\mathrm{f,glo}}Z_{\mathrm{f,glo}}(t-k).
\end{aligned} \tag{2}$$

Similarly for the slow component, $\epsilon_{\mathrm{iv,s,glo}}(t) = \sum_{k=0}^{\infty} \alpha^k_{\mathrm{s,glo}}Z_{\mathrm{s,glo}}(t-k)$.

The variance of $\epsilon_{\mathrm{iv,f,glo}}(t)$ is therefore:

$$
\begin{aligned}
\mathrm{Var}[\epsilon_{\mathrm{iv,f,glo}}(t)] &= \mathrm{Var}\left[\sum_{k=0}^{\infty} \alpha_{\mathrm{f,glo}}^{k} Z_{\mathrm{f,glo}}(t-k)\right], \\
&= \sum_{k=0}^{\infty} \mathrm{Var}[\alpha_{\mathrm{f,glo}}^{k} Z_{\mathrm{f,glo}}(t-k)], \\
&= \sum_{k=0}^{\infty} \alpha_{\mathrm{f,glo}}^{2k}\, \mathrm{Var}[Z_{\mathrm{f,glo}}(t-k)], \\
&= \sum_{k=0}^{\infty} \alpha_{\mathrm{f,glo}}^{2k} \sigma_{\mathrm{f,glo}}^{2}, \\
&= \sigma_{\mathrm{f,glo}}^{2} \sum_{k=0}^{\infty} \alpha_{\mathrm{f,glo}}^{2k}, \\
&= \frac{\sigma_{\mathrm{f,glo}}^{2}}{1-\alpha_{\mathrm{f,glo}}^{2}}.
\end{aligned} \tag{3}
$$

Similarly for the slow component, $\mathrm{Var}[\epsilon_{\mathrm{iv,s,glo}}(t)] = \dfrac{\sigma_{\mathrm{s,glo}}^{2}}{1-\alpha_{\mathrm{s,glo}}^{2}}$.

The autocovariance function at lag $h > 0$ of $\boldsymbol{\epsilon}_{\mathrm{iv,f,glo}}$ is calculated as follows:

$$
\begin{aligned}
\mathrm{Cov}[\epsilon_{\mathrm{iv,f,glo}}(t), \epsilon_{\mathrm{iv,f,glo}}(t+h)] &= \mathbb{E}[\epsilon_{\mathrm{iv,f,glo}}(t)\epsilon_{\mathrm{iv,f,glo}}(t+h)] - \mathbb{E}[\epsilon_{\mathrm{iv,f,glo}}(t)]\, \mathbb{E}[\epsilon_{\mathrm{iv,f,glo}}(t+h)], \\
&= \mathbb{E}[\epsilon_{\mathrm{iv,f,glo}}(t)\epsilon_{\mathrm{iv,f,glo}}(t+h)], \\
&= \mathbb{E}\left[\left(\sum_{k=0}^{\infty} \alpha_{\mathrm{f,glo}}^{k} Z_{\mathrm{f,glo}}(t-k)\right)\left(\sum_{j=0}^{\infty} \alpha_{\mathrm{f,glo}}^{j} Z_{\mathrm{f,glo}}(t+h-j)\right)\right], \\
&= \sum_{k=0}^{\infty}\sum_{j=0}^{\infty} \alpha_{\mathrm{f,glo}}^{k} \alpha_{\mathrm{f,glo}}^{j} \underbrace{\mathbb{E}[Z_{\mathrm{f,glo}}(t-k) Z_{\mathrm{f,glo}}(t+h-j)]}_{=0\ \forall\ j \neq k+h}, \\
&= \sum_{k=0}^{\infty} \alpha_{\mathrm{f,glo}}^{k} \alpha_{\mathrm{f,glo}}^{k+h} \sigma_{\mathrm{f,glo}}^{2}, \\
&= \sigma_{\mathrm{f,glo}}^{2} \alpha_{\mathrm{f,glo}}^{h} \sum_{k=0}^{\infty} \alpha_{\mathrm{f,glo}}^{2k}, \\
&= \frac{\sigma_{\mathrm{f,glo}}^{2}}{1-\alpha_{\mathrm{f,glo}}^{2}} \alpha_{\mathrm{f,glo}}^{h}.
\end{aligned} \tag{4}
$$

Similarly for the slow component, $\mathrm{Cov}[\epsilon_{\mathrm{iv,s,glo}}(t), \epsilon_{\mathrm{iv,s,glo}}(t+h)] = \dfrac{\sigma_{\mathrm{s,glo}}^{2}}{1-\alpha_{\mathrm{s,glo}}^{2}} \alpha_{\mathrm{s,glo}}^{h}$.

The slow and fast components within each residual are independent:

$$\begin{cases} \text{Cov}[\epsilon_{\text{iv,f,glo}}(t), \epsilon_{\text{iv,s,glo}}(t)] & = 0, \\ \text{Cov}[\epsilon_{\text{iv,f,loc}}(t), \epsilon_{\text{iv,s,loc}}(t)] & = 0. \end{cases} \tag{5}$$

Therefore, the coefficients $\Sigma_{\text{iv,glo},ij}$ and $\Sigma_{\text{iv,loc},ij}$, i.e. the covariances at lag $|i-j|$ with $(i,j) \in [\![1, n_y]\!]^2$ are given by:

$$\begin{cases} \Sigma_{\text{iv,loc},ij} = \frac{\sigma^2_{\text{f,loc}}}{1-\alpha^2_{\text{f,loc}}} \alpha^{|i-j|}_{\text{f,loc}} + \frac{\sigma^2_{\text{s,loc}}}{1-\alpha^2_{\text{s,loc}}} \alpha^{|i-j|}_{\text{s,loc}}, \\ \Sigma_{\text{iv,glo},ij} = \frac{\sigma^2_{\text{f,glo}}}{1-\alpha^2_{\text{f,glo}}} \alpha^{|i-j|}_{\text{f,glo}} + \frac{\sigma^2_{\text{s,glo}}}{1-\alpha^2_{\text{s,glo}}} \alpha^{|i-j|}_{\text{s,glo}}. \end{cases} \tag{6}$$

In a matrix form, $\mathbf{\Sigma}_{\text{iv,glo}}$ and $\mathbf{\Sigma}_{\text{iv,loc}}$ write:

$$\mathbf{\Sigma}_{\text{iv,loc}} = \frac{\sigma^2_{\text{f,loc}}}{1-\alpha^2_{\text{f,loc}}} \begin{bmatrix} 1 & \alpha_{\text{f,loc}} & \cdots & \alpha^{n_y-1}_{\text{f,loc}} \\ \alpha_{\text{f,loc}} & \ddots & \ddots & \\ \vdots & \ddots & \ddots & \alpha_{\text{f,loc}} \\ \alpha^{n_y-1}_{\text{f,loc}} & & \alpha_{\text{f,loc}} & 1 \end{bmatrix} + \frac{\sigma^2_{\text{s,loc}}}{1-\alpha^2_{\text{s,loc}}} \begin{bmatrix} 1 & \alpha_{\text{s,loc}} & \cdots & \alpha^{n_y-1}_{\text{s,loc}} \\ \alpha_{\text{s,loc}} & \ddots & \ddots & \\ \vdots & \ddots & \ddots & \alpha_{\text{s,loc}} \\ \alpha^{n_y-1}_{\text{s,loc}} & & \alpha_{\text{s,loc}} & 1 \end{bmatrix}, \tag{7}$$

$$\mathbf{\Sigma}_{\text{iv,glo}} = \frac{\sigma^2_{\text{f,glo}}}{1-\alpha^2_{\text{f,glo}}} \begin{bmatrix} 1 & \alpha_{\text{f,glo}} & \cdots & \alpha^{n_y-1}_{\text{f,glo}} \\ \alpha_{\text{f,glo}} & \ddots & \ddots & \\ \vdots & \ddots & \ddots & \alpha_{\text{f,glo}} \\ \alpha^{n_y-1}_{\text{f,glo}} & & \alpha_{\text{f,glo}} & 1 \end{bmatrix} + \frac{\sigma^2_{\text{s,glo}}}{1-\alpha^2_{\text{s,glo}}} \begin{bmatrix} 1 & \alpha_{\text{s,glo}} & \cdots & \alpha^{n_y-1}_{\text{s,glo}} \\ \alpha_{\text{s,glo}} & \ddots & \ddots & \\ \vdots & \ddots & \ddots & \alpha_{\text{s,glo}} \\ \alpha^{n_y-1}_{\text{s,glo}} & & \alpha_{\text{s,glo}} & 1 \end{bmatrix}. \tag{8}$$

Estimation of $\mathbf{\Sigma}_{\text{iv,dep}}$

To model the dependence between internal variability within the GMST and local internal variability in the covariance matrix $\mathbf{\Sigma}_{\text{iv,dep}}$, we need to calculate the covariances $\text{Cov}[\epsilon_{\text{s,glo}}(t), \epsilon_{\text{s,loc}}(t)]$ and $\text{Cov}[\epsilon_{\text{f,glo}}(t), \epsilon_{\text{f,loc}}(t)]$. We can write:

$$\begin{bmatrix} \epsilon_{\text{f,glo}}(t) \\ \epsilon_{\text{f,loc}}(t) \end{bmatrix} = \begin{bmatrix} \alpha_{\text{f,glo}} & 0 \\ 0 & \alpha_{\text{f,glo}} \end{bmatrix} \begin{bmatrix} \epsilon_{\text{f,glo}}(t-1) \\ \epsilon_{\text{f,loc}}(t-1) \end{bmatrix} + \begin{bmatrix} Z_{\text{f,glo}}(t) \\ Z_{\text{f,loc}}(t) \end{bmatrix}. \tag{9}$$

This vector autoregressive model can also be written:

$$\boldsymbol{\epsilon_{f,t}} = \boldsymbol{A}\boldsymbol{\epsilon_{f,t-1}} + \boldsymbol{Z_{f,t}}, \tag{10}$$

where $\boldsymbol{\epsilon_{f,t}}$ and $\boldsymbol{Z_{f,t}}$ are vectors and $\boldsymbol{A}$ is a matrix.

Computing the covariance of $\boldsymbol{\epsilon_{f,t}}$ gives:

$$\boldsymbol{\Sigma_{\epsilon}} = \boldsymbol{A}\boldsymbol{\Sigma_{\epsilon}}\boldsymbol{A}' + \boldsymbol{\Sigma_{Z}}, \tag{11}$$

where $\boldsymbol{\Sigma_{\epsilon}} = \text{Cov}[\boldsymbol{\epsilon_{f,t}}]$ and $\boldsymbol{\Sigma_{Z}} = \text{Cov}[\boldsymbol{Z_{f,t}}]$. As we want to allow a relationship between the local and global fast components, we slightly change the distribution parameters associated of the white noises, and introduce a parameter λ, which links $Z_{\text{f,loc}}(t)$ to $Z_{\text{f,glo}}(t)$ in $\boldsymbol{\Sigma_{Z}}$ such as:

$$\boldsymbol{\Sigma_{Z}} = \begin{bmatrix} \dfrac{\lambda^2\sigma^2_{\text{f,loc}}}{1-\alpha^2_{\text{f,loc}}} & \dfrac{\lambda\sigma_{\text{f,loc}}\sigma_{\text{f,glo}}}{\sqrt{1-\alpha^2_{\text{f,loc}}}\sqrt{1-\alpha^2_{\text{f,glo}}}} \\ \dfrac{\lambda\sigma_{\text{f,glo}}\sigma_{\text{f,loc}}}{\sqrt{1-\alpha^2_{\text{f,glo}}}\sqrt{1-\alpha^2_{\text{f,loc}}}} & \dfrac{\lambda^2\sigma^2_{\text{f,glo}}}{1-\alpha^2_{\text{f,glo}}} \end{bmatrix}. \tag{12}$$

Coefficients of $\boldsymbol{\Sigma_{\epsilon}}$ are given by solving equation 11:

$$\text{Vec}(\boldsymbol{\Sigma_{\epsilon}}) = [\boldsymbol{I} - \boldsymbol{A} \otimes \boldsymbol{A}']^{-1}\text{Vec}(\boldsymbol{\Sigma_{Z}}), \tag{13}$$

where Vec stands for the vectorisation and $\otimes$ for the Kronecker product. Equation 13 can be written:

$$\begin{bmatrix} \text{Var}[\epsilon_{\text{f,glo}}(t)] \\ \text{Cov}[\epsilon_{\text{f,glo}}(t), \epsilon_{\text{f,loc}}(t)] \\ \text{Cov}[\epsilon_{\text{f,loc}}(t), \epsilon_{\text{f,glo}}(t)] \\ \text{Var}[\epsilon_{\text{f,loc}}(t)] \end{bmatrix} = \begin{bmatrix} 1-\alpha_{\text{f,glo}}^2 & 0 & 0 & 0 \\ 0 & 1-\alpha_{\text{f,glo}}\alpha_{\text{f,loc}} & 0 & 0 \\ 0 & 0 & 1-\alpha_{\text{f,loc}}\alpha_{\text{f,glo}} & 0 \\ 0 & 0 & 0 & 1-\alpha_{\text{f,loc}}^2 \end{bmatrix}^{-1} \begin{bmatrix} \frac{\lambda^2\sigma_{\text{f,loc}}^2}{1-\alpha_{\text{f,loc}}^2} \\ \frac{\lambda\sigma_{\text{f,loc}}\sigma_{\text{f,glo}}}{\sqrt{1-\alpha_{\text{f,loc}}^2}\sqrt{1-\alpha_{\text{f,glo}}^2}} \\ \frac{\lambda\sigma_{\text{f,glo}}\sigma_{\text{f,loc}}}{\sqrt{1-\alpha_{\text{f,glo}}^2}\sqrt{1-\alpha_{\text{f,loc}}^2}} \\ \frac{\lambda^2\sigma_{\text{f,glo}}^2}{1-\alpha_{\text{f,glo}}^2} \end{bmatrix} \tag{14}$$

Then we obtain:

$$\text{Cov}[\epsilon_{\text{f,glo}}(t), \epsilon_{\text{f,loc}}(t)] = \frac{\lambda\sigma_{\text{f,glo}}\sigma_{\text{f,loc}}}{\sqrt{1-\alpha_{\text{f,glo}}^2}\sqrt{1-\alpha_{\text{f,loc}}^2}(1-\alpha_{\text{f,glo}}\alpha_{\text{f,loc}})}. \tag{15}$$

The covariance at lag $h > 0$ between $\epsilon_{\text{f,glo}}(t)$ and $\epsilon_{\text{f,loc}}(t-h)$ is calculated using back substitutions:

$$\begin{aligned} \text{Cov}[\epsilon_{\text{f,glo}}(t), \epsilon_{\text{f,loc}}(t-h)] &= \mathbb{E}[\epsilon_{\text{f,glo}}(t)\epsilon_{\text{f,loc}}(t-h)], \\ &= \mathbb{E}[\alpha_{\text{f,glo}}\epsilon_{\text{f,glo}}(t-1)\epsilon_{\text{f,loc}}(t-h)] + \underbrace{\mathbb{E}[Z_{f,glo}(t)(\epsilon_{\text{f,loc}}(t-h))]}_{=0}, \\ &= \alpha_{\text{f,glo}}\,\text{Cov}[\epsilon_{\text{f,glo}}(t-1), \epsilon_{\text{f,loc}}(t-h)], \\ &= \vdots \\ &= \alpha_{\text{f,glo}}^h\,\text{Cov}[\epsilon_{\text{f,glo}}(t-h), \epsilon_{\text{f,loc}}(t-h)], \\ &= \alpha_{\text{f,glo}}^h \frac{\lambda\sigma_{\text{f,glo}}\sigma_{\text{f,loc}}}{\sqrt{1-\alpha_{\text{f,glo}}^2}\sqrt{1-\alpha_{\text{f,loc}}^2}(1-\alpha_{\text{f,glo}}\alpha_{\text{f,loc}})}. \end{aligned} \tag{16}$$

Similarly, we can write the covariance at lag $h > 0$ between $\epsilon_{\text{f,glo}}(t-h)$ and $\epsilon_{\text{f,loc}}(t)$:

$$\text{Cov}[\epsilon_{\text{f,glo}}(t-h), \epsilon_{\text{f,loc}}(t)] = \alpha_{\text{f,loc}}^h \frac{\lambda\sigma_{\text{f,glo}}\sigma_{\text{f,loc}}}{\sqrt{1-\alpha_{\text{f,glo}}^2}\sqrt{1-\alpha_{\text{f,loc}}^2}(1-\alpha_{\text{f,glo}}\alpha_{\text{f,loc}})}. \tag{17}$$

The same calculations applied to the slow components give the following covariances:

$$\begin{cases} \mathrm{Cov}[\epsilon_{\mathrm{s,glo}}(t-h), \epsilon_{\mathrm{s,loc}}(t)] & = \alpha_{\mathrm{s,loc}}^{h} \dfrac{\lambda \sigma_{\mathrm{s,glo}} \sigma_{\mathrm{s,loc}}}{\sqrt{1-\alpha_{\mathrm{s,glo}}^{2}}\sqrt{1-\alpha_{\mathrm{s,loc}}^{2}}(1-\alpha_{\mathrm{s,glo}}\alpha_{\mathrm{s,loc}})}, \\ \mathrm{Cov}[\epsilon_{\mathrm{s,glo}}(t), \epsilon_{\mathrm{s,loc}}(t-h)] & = \alpha_{\mathrm{s,glo}}^{h} \dfrac{\lambda \sigma_{\mathrm{s,glo}} \sigma_{\mathrm{s,loc}}}{\sqrt{1-\alpha_{\mathrm{s,glo}}^{2}}\sqrt{1-\alpha_{\mathrm{s,loc}}^{2}}(1-\alpha_{\mathrm{s,glo}}\alpha_{\mathrm{s,loc}})}. \end{cases} \tag{18}$$

Therefore, $\mathbf{\Sigma}_{\mathrm{iv,dep}}$ takes the form of a Toeplitz matrix:

$$\mathbf{\Sigma}_{\mathrm{iv,dep}} = C_f \begin{bmatrix} 1 & \alpha_{\mathrm{f,loc}} & \dots & \alpha_{\mathrm{f,loc}}^{n_y-1} \\ \alpha_{\mathrm{f,glo}} & \ddots & \ddots & \\ \vdots & \ddots & \ddots & \alpha_{\mathrm{f,loc}} \\ \alpha_{\mathrm{f,glo}}^{n_y-1} & & \alpha_{\mathrm{f,glo}} & 1 \end{bmatrix} + C_s \begin{bmatrix} 1 & \alpha_{\mathrm{s,loc}} & \dots & \alpha_{\mathrm{s,loc}}^{n_y-1} \\ \alpha_{\mathrm{s,glo}} & \ddots & \ddots & \\ \vdots & \ddots & \ddots & \alpha_{\mathrm{s,loc}} \\ \alpha_{\mathrm{s,glo}}^{n_y-1} & & \alpha_{\mathrm{s,glo}} & 1 \end{bmatrix}, \tag{19}$$

where $C_f = \mathrm{Cov}[\epsilon_{\mathrm{f,glo}}(t), \epsilon_{\mathrm{f,loc}}(t)]$ and $C_s = \mathrm{Cov}[\epsilon_{\mathrm{s,glo}}(t), \epsilon_{\mathrm{s,loc}}(t)]$.

As the correlation between local and global internal variability depends on the considered location, λ is not set to a fixed value for all the grid points. Instead, for each $\epsilon_{\mathrm{iv,loc}}$, we bound λ between -1 and 1 by considering the ratio between the empirical correlation and the correlation associated with our MAR models. In practice, λ is estimated by using a method of moments:

$$\lambda = \begin{cases} -1, & \text{if } \frac{\rho_{\mathrm{res}}}{\rho_{\mathrm{mar}}} < -1, \\ 1, & \text{if } \frac{\rho_{\mathrm{res}}}{\rho_{\mathrm{mar}}} > 1, \\ \frac{\rho_{\mathrm{res}}}{\rho_{\mathrm{mar}}}, & \text{otherwise}, \end{cases} \tag{20}$$

where ρ_{res} is the correlation between the local and global observed residuals, and $\rho_{\mathrm{mar}} = \dfrac{C_{\mathrm{f,max}} + C_{\mathrm{s,max}}}{\sqrt{(\sigma_{\mathrm{f,loc}} + \sigma_{\mathrm{s,loc}})(\sigma_{\mathrm{f,glo}} + \sigma_{\mathrm{s,glo}})}}$ is the correlation based on the MAR parameters previously estimated from equations 7 and 8. $C_{\mathrm{f,max}}$ ($C_{\mathrm{s,max}}$) corresponds to the case of a total dependence between $\epsilon_{\mathrm{f,glo}}(t)$ and $\epsilon_{\mathrm{f,loc}}(t)$ ($\epsilon_{\mathrm{s,glo}}(t)$ and $\epsilon_{\mathrm{s,loc}}(t)$), i.e. to the covariance C_f (C_s) with $\lambda = 1$.

The spatial pattern of λ is shown in Fig. S31. Overall, our statistical model remarkably captures the dependency between local and global residuals, especially over the tropical Pacific and Atlantic regions.

Structure of the observation operator $\boldsymbol{H}$

The observation operator $\boldsymbol{H}$ is a matrix of size $2n_y \times 2n_x$, where n_y (n_x) is the number of observed (simulated) years, *i.e.* 170 (251) which is the length of the vectors $\boldsymbol{y}_{\text{loc}}$ et $\boldsymbol{y}_{\text{glo}}$ ($\boldsymbol{x}_{\text{loc}}$ and $\boldsymbol{x}_{\text{glo}}$). When $\boldsymbol{x} = (\boldsymbol{x}_{\text{loc}}, \boldsymbol{x}_{\text{glo}})$ is solely constrained by GMST observations, *i.e.* only by $\boldsymbol{y}_{\text{glo}}$, $\boldsymbol{H}$ extracts $\boldsymbol{x}_{\text{glo}}$ from $\boldsymbol{x}$ over the observed 1850-2021 period. To do so, the square submatrix $\boldsymbol{H}_{\text{glo}}$ of $\boldsymbol{H}$ (see equation 21) is the identity matrix; $\boldsymbol{H}_{\text{loc}}$ (see equation 21) and all other coefficients equal zero. Constraining by both GMST and local observations consists in setting the submatrix $\boldsymbol{H}_{\text{loc}}$ and $\boldsymbol{H}_{\text{glo}}$ equal to identity and all other coefficients equal to zero.

$$\boldsymbol{H} = \begin{bmatrix} \overbrace{\boldsymbol{H}_{\text{loc}}}^{n_y} & \begin{matrix} 0 \\ \vdots \\ 0 \end{matrix} & \overbrace{\begin{matrix} 0 & \cdots & \cdots & 0 \\ \vdots & & & \vdots \\ 0 & \cdots & \cdots & 0 \end{matrix}}^{n_x} \\ \underbrace{\begin{matrix} 0 & \cdots & \cdots & 0 \\ \vdots & & & \vdots \\ 0 & \cdots & \cdots & 0 \end{matrix}}_{n_x} & & \underbrace{\boldsymbol{H}_{\text{glo}}}_{n_y} \quad \begin{matrix} 0 \\ \vdots \\ 0 \end{matrix} \end{bmatrix} \tag{21}$$

Calculation of $p(\boldsymbol{x}|\boldsymbol{y})$

Equation 4 from the Methods section in the main text can also be written in matrix form:

$$\begin{bmatrix} \boldsymbol{x} \\ \boldsymbol{y} \end{bmatrix} = \begin{bmatrix} \boldsymbol{I} & \boldsymbol{0} \\ \boldsymbol{0} & \boldsymbol{H} \end{bmatrix} \begin{bmatrix} \boldsymbol{x} \\ \boldsymbol{x} \end{bmatrix} + \begin{bmatrix} 0 \\ \epsilon \end{bmatrix} \sim \mathcal{N}\left(\begin{bmatrix} \boldsymbol{\mu} \\ \boldsymbol{H}\boldsymbol{\mu} \end{bmatrix}, \begin{bmatrix} \boldsymbol{\Sigma}_{\text{mod}} & \boldsymbol{\Sigma}_{\text{mod}}\boldsymbol{H}' \\ \boldsymbol{H}\boldsymbol{\Sigma}_{\text{mod}} & \boldsymbol{H}\boldsymbol{\Sigma}_{\text{mod}}\boldsymbol{H}' + \boldsymbol{\Sigma}_{\text{obs}} \end{bmatrix} \right), \tag{22}$$

This formulation allows to easily derive $\boldsymbol{x}|\boldsymbol{y}$ with the Gaussian conditioning theorem. Thus, the

posterior $p(\boldsymbol{x}|\boldsymbol{y})$ is written as follows:

$$p(\boldsymbol{x}|\boldsymbol{y}=\boldsymbol{y_0}) \sim \mathcal{N}(\boldsymbol{\mu}+\boldsymbol{\Sigma}_{\text{mod}}\boldsymbol{H}'(\boldsymbol{H}\boldsymbol{\Sigma}_{\text{mod}}\boldsymbol{H}'+\boldsymbol{\Sigma}_{\text{obs}})^{-1}(\boldsymbol{y_o}-\boldsymbol{H}\boldsymbol{\mu}), \boldsymbol{\Sigma}_{\text{mod}}-\boldsymbol{\Sigma}_{\text{mod}}\boldsymbol{H}'(\boldsymbol{H}\boldsymbol{\Sigma}_{\text{mod}}\boldsymbol{H}'+\boldsymbol{\Sigma}_{\text{obs}})^{-1}\boldsymbol{H}\boldsymbol{\Sigma}_{\text{mod}}). \tag{23}$$

Supplementary Discussion

The coverage probability is used as a second criterion to assess the performance of the method, and quantifies the probability that the constrained confidence range contains the true value. Results suggest that the KCC method is reliable in the GMST-only case. For example, the estimate of the coverage probability is 89% for the European point (number 1 on Fig. 5) over the 2021-2040 period, which is very close to the expected nominal level (90%, since all confidence ranges are 5–95%) (Fig. S3). Furthermore, the method is able to correctly predict values which lie near the boundary or even outside the assumed 5-95% prior uncertainty range (eg, GFDL-ESM4 and CanESM5 models in Fig. S3). When all regions are considered, the median of the coverage probability distribution is 88% (Fig. S4), with half of the values lying between 85% and 91%, which is fairly reasonable given the uncertainty in its estimation (see description of Fig. S3).

In the Local+GMST case, the addition of local pseudo-observations leads to a further reduction in uncertainty ranges. The median of the estimated coverage probabilities is higher than 85% and so remains consistent with, or close to its nominal level of 90%.

Note that the three constrained methods lead to systematically reduced coverage probability while, ideally, we should obtain coverage values equal to 90%. Here, this threshold is hardly reached for two reasons. First, even in the unconstrained case, the median coverage is lower than 90%. This could be explained by a high/low sensitivity of some models to greenhouse gases in the projections compared to what is predicted by other (unconstrained) models. This also affects the constrained distribution, contributing to coverage values below 90%. This could be a matter for further research. Second, the KCC method can be deficient by not being able to predict the true value while it is compatible with the unconstrained projections.

To better understand and interpret the coverage probabilities, we calculated the confusion matrix, counting the number of cases for which the true value (from the pseudo-obs) :

- is included in both the constrained range $p(\boldsymbol{x}|\boldsymbol{y})$ and the unconstrained range $\Pi(\boldsymbol{x})$ (true positive rate),
- not included in both the constrained and unconstrained ranges (true negative rate),
- is included in the constrained range, but not in the unconstrained range (false positive rate),
- is included in the unconstrained range, but not in the constrained range (false negative rate).

Unlike a classical confusion matrix, we consider the false positive rate (usually qualified as a false alarm rate) as an added value of the method, since it is able to predict the true value while the unconstrained projections are not. The rates are shown in Table , accounting for the 121 pseudo-observations and 55 locations. The sum of the true positive, and false positive rates (associated with improvement cases) indicates that the method is reliable in 85, 84 and 88% of the cases for constraints based on Local-only, Local+GMST and GMST-only observations. The decrease in coverage probabilities is partly due to the true negative rates (total number of cases where the true value is not included in the range) which account for about 5%. We consider a miss rate between 7 and 11% as quite acceptable (recall that that $p(\boldsymbol{x}|\boldsymbol{y})$ and $\Pi(\boldsymbol{x})$ are distributions that are assessed by 5-95% ranges).

	Local-only		Local+GMST		GMST-only	
True value from pseudo-obs	Included in $p(\boldsymbol{x}\|\boldsymbol{y})$	Not included in $p(\boldsymbol{x}\|\boldsymbol{y})$	Included in $p(\boldsymbol{x}\|\boldsymbol{y})$	Not included in $p(\boldsymbol{x}\|\boldsymbol{y})$	Included in $p(\boldsymbol{x}\|\boldsymbol{y})$	Not included in $p(\boldsymbol{x}\|\boldsymbol{y})$
Included in $\Pi(\boldsymbol{x})$	80	9	78	11	82	7
Not included in $\Pi(\boldsymbol{x})$	5	6	6	5	6	5

Table S 1: **Confusion matrix relating the temperature projection constrained by the KCC method and the true value from pseudo-observations.** Rates (in %) are computed from 6776 cases (121 pseudo-observations and 55 locations). Each rate is normalised by the number of ensemble members for each model to avoid giving too much weight to models with a large ensemble.

Consistent with the CRPSS, excluding the models characterised by a strong low-frequency variability leads to an improvement of the coverage probabilities. The distribution gets slightly closer to the 90% level (see Fig. S19).

Supplementary Figures

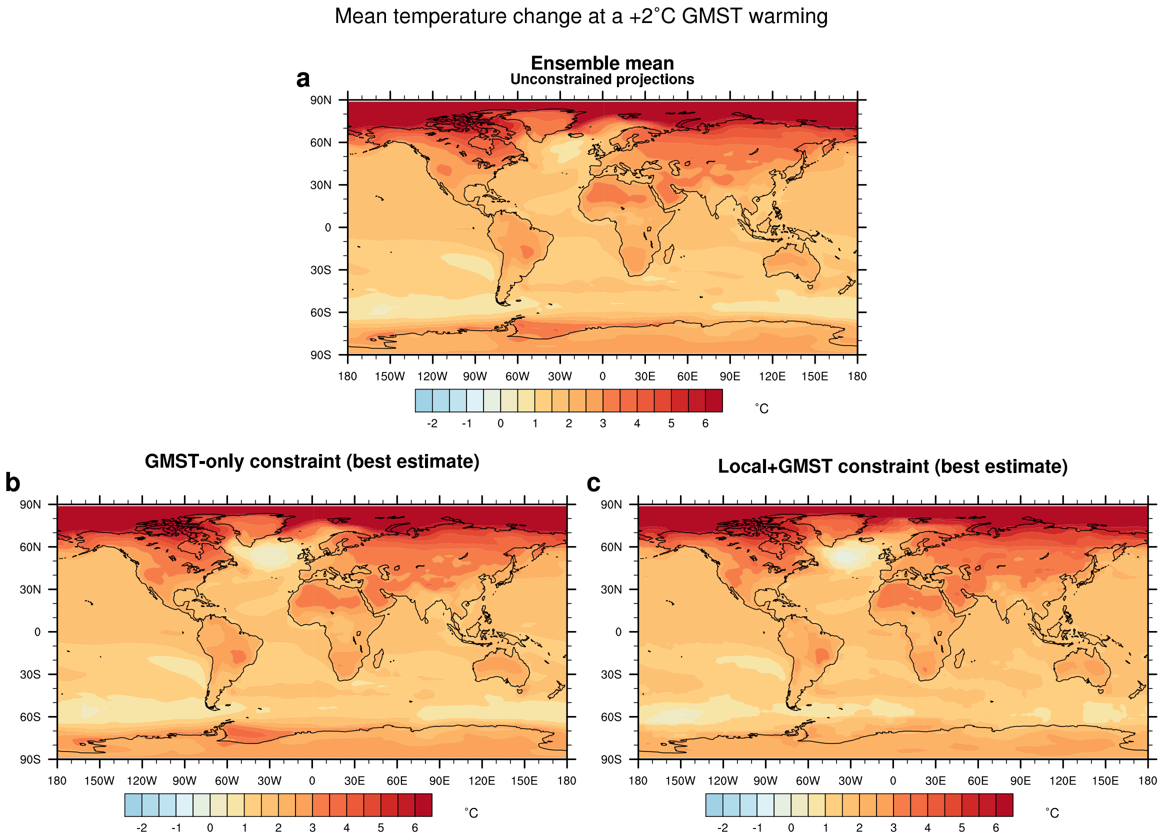

Fig. S 1: **Warming pattern at +2 °C of GMST warming**. (a) Ensemble mean of the unconstrained local temperature changes. (b) Best estimate of the constrained local temperature changes in the GMST-only case. (c) Same as (b) but for the Local+GMST case. All values in (a), (b) and (c) are anomalies with respect to the 1850-1900 period.

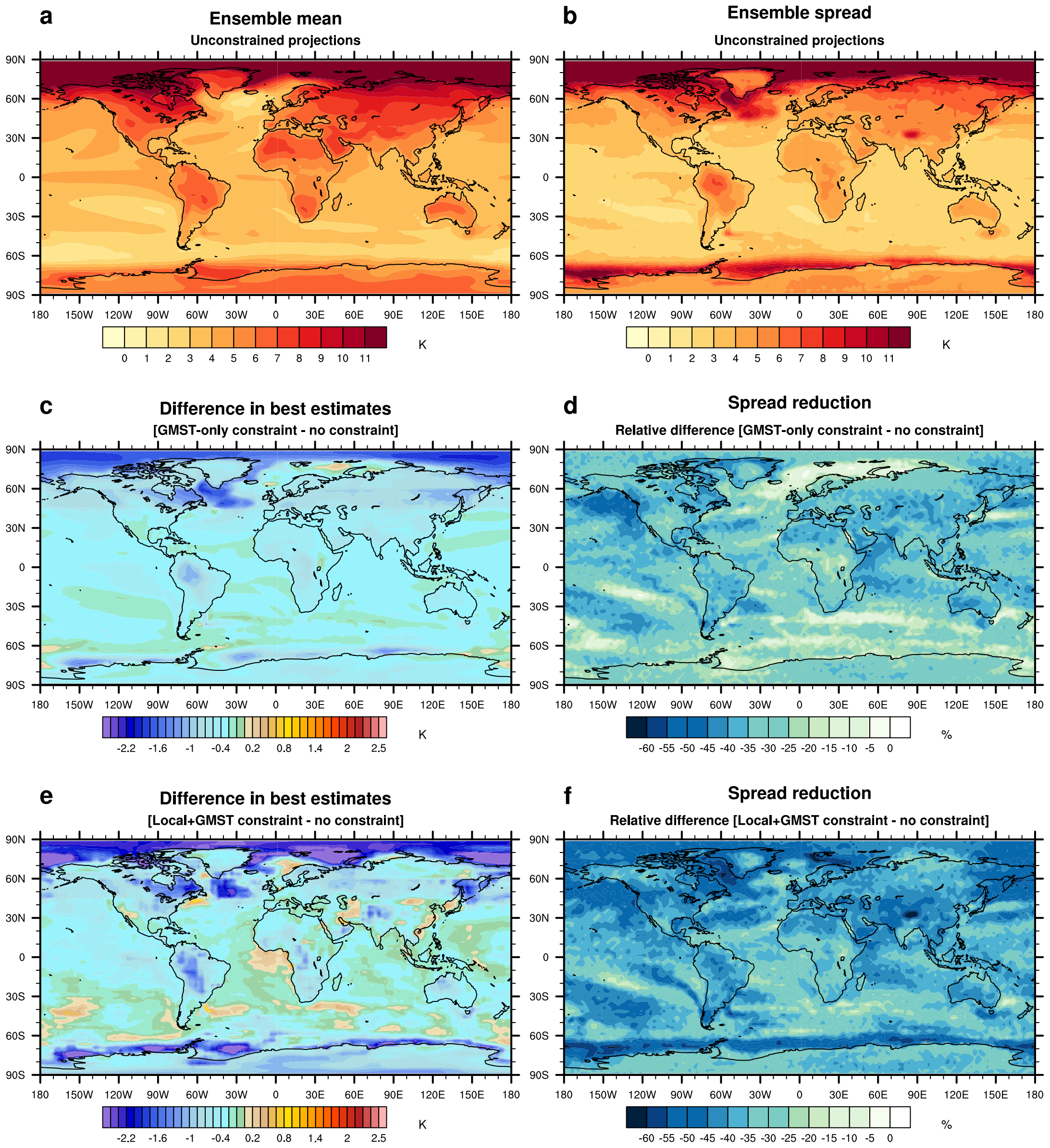

Fig. S 2: Same as Fig. 3 but for the 2081-2100 period. Note that the scale in the color bars are different.

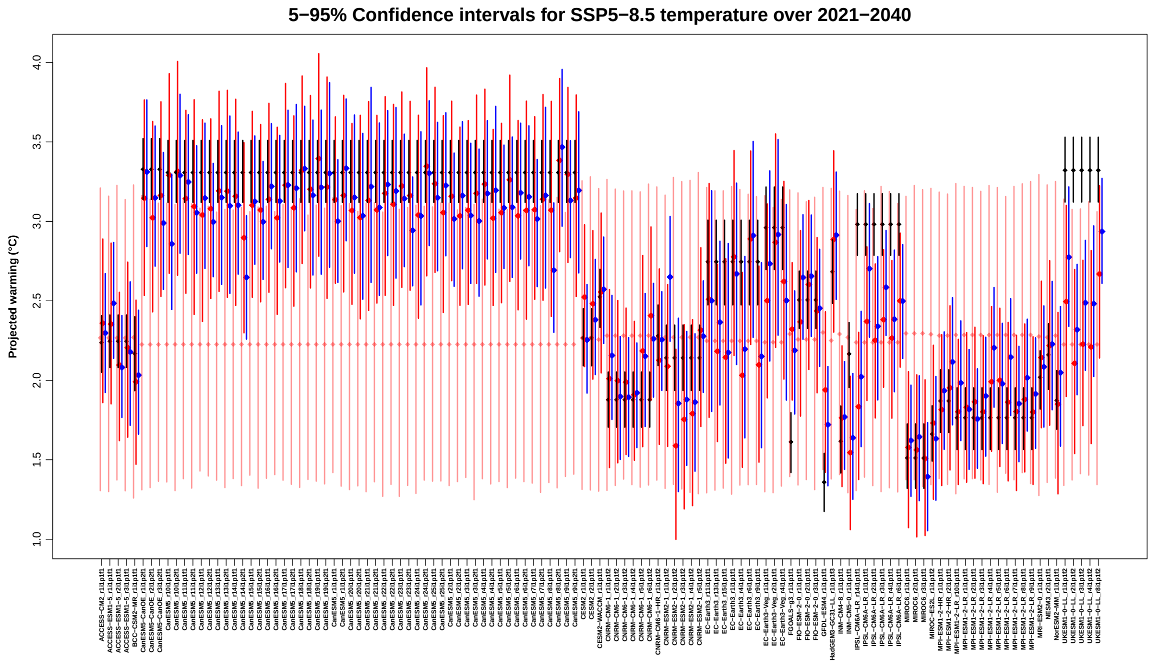

Fig. S 3: **Coverage probabilities of the constraining method applied for the local temperature over a European grid point within a perfect model framework.** 121 concatenated simulations of historical + SSP5-8.5 scenarios are considered. Each of these provide pseudo-observations over the historical period (1850–2021) that are used to constrain temperature projections and to infer the 2021–2040 warming under this scenario. The effective warming, as estimated from the model ensemble mean after applying temporal smoothing, is shown in black (with 5–95% uncertainty). The unconstrained distribution derived from all CMIP6 models but the one used in pseudo-observations is shown in pink. The constrained distribution obtained in the GMST-only (Local+GMST) case is shown in red (blue). All values are anomalies with respect to the 1850–1900 period. Note that for all pseudo-observations of a given model (eg, the 50 members of the CanESM5 model), the same prior distribution is derived from all other models, so pink ranges should be identical. Small differences in this case arise because a sample of 1000 realizations is drawn from the prior distribution to produce this plot. This phenomenon can induce some uncertainty in the estimation of the coverage probabilities (not quantified here).

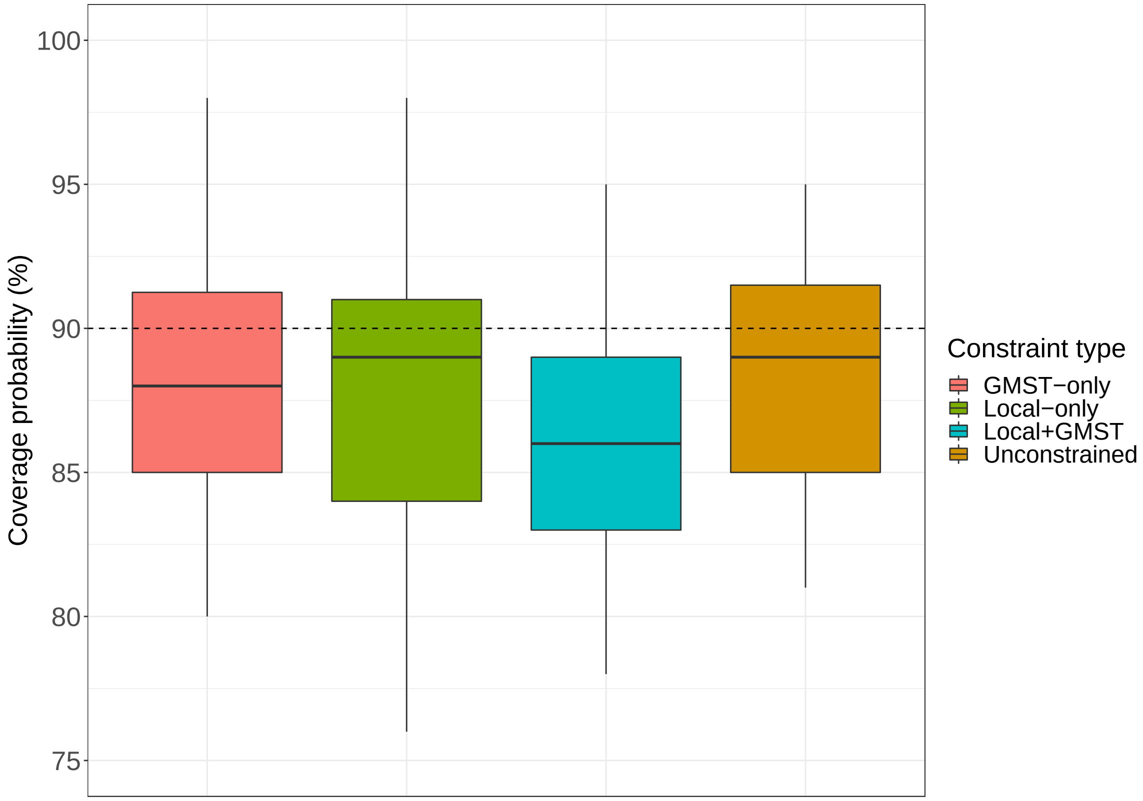

Fig. S 4: **Coverage probabilities of the constraining method applied for the local temperature within a perfect model framework.** The red, green, blue and orange boxplots indicate the coverage probability distributions in the GMST-only, Local-only, Local+GMST and unconstrained cases, respectively, for all locations (see points in Fig. 5) and models over the 2021-2040 period. The top (bottom) of the box represents the 25th (75th) percentile of the distribution and the upper (lower) whisker represents the 5th (95th) percentile. Values are normalised by the number of members in each model.

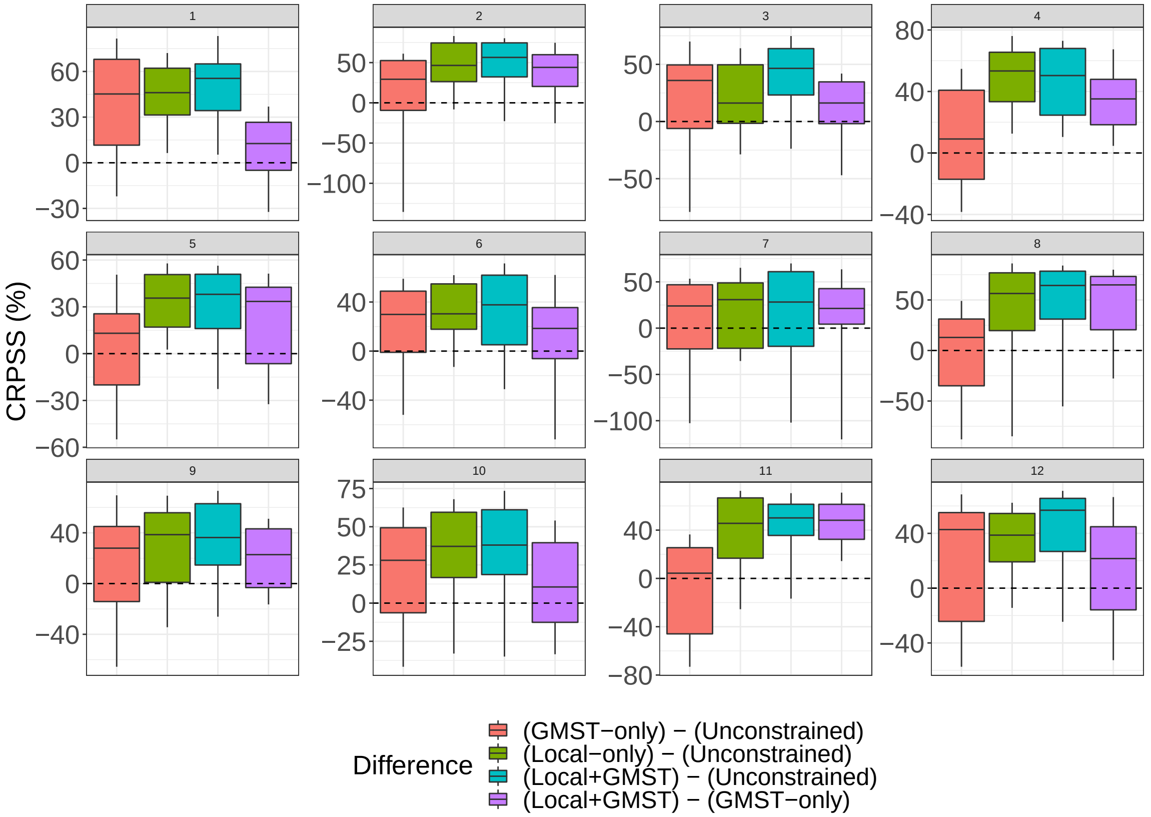

Fig. S 5: **CRPSS for the constrained temperature projections for every location within the perfect model framework.** The red, green and blue boxplots (one per location) indicate the CRPSS distributions in the GMST-only, Local-only and Local+GMST cases, respectively, compared to the unconstrained case for all locations (see points in Fig. 5) and models over the 2021-2040 period. The boxplot in magenta indicates the added value from the local observations and stand for the CRPSS distributions in the Local+GMST case compared to the GMST-only case. The top (bottom) of the box represents the 25th (75th) percentile of the distribution and the upper (lower) whisker represents the 5th (95th) percentile. Values are normalised by the number of members in each model. A CRPSS of 0 (dashed line) indicates the absence of added value of the method.

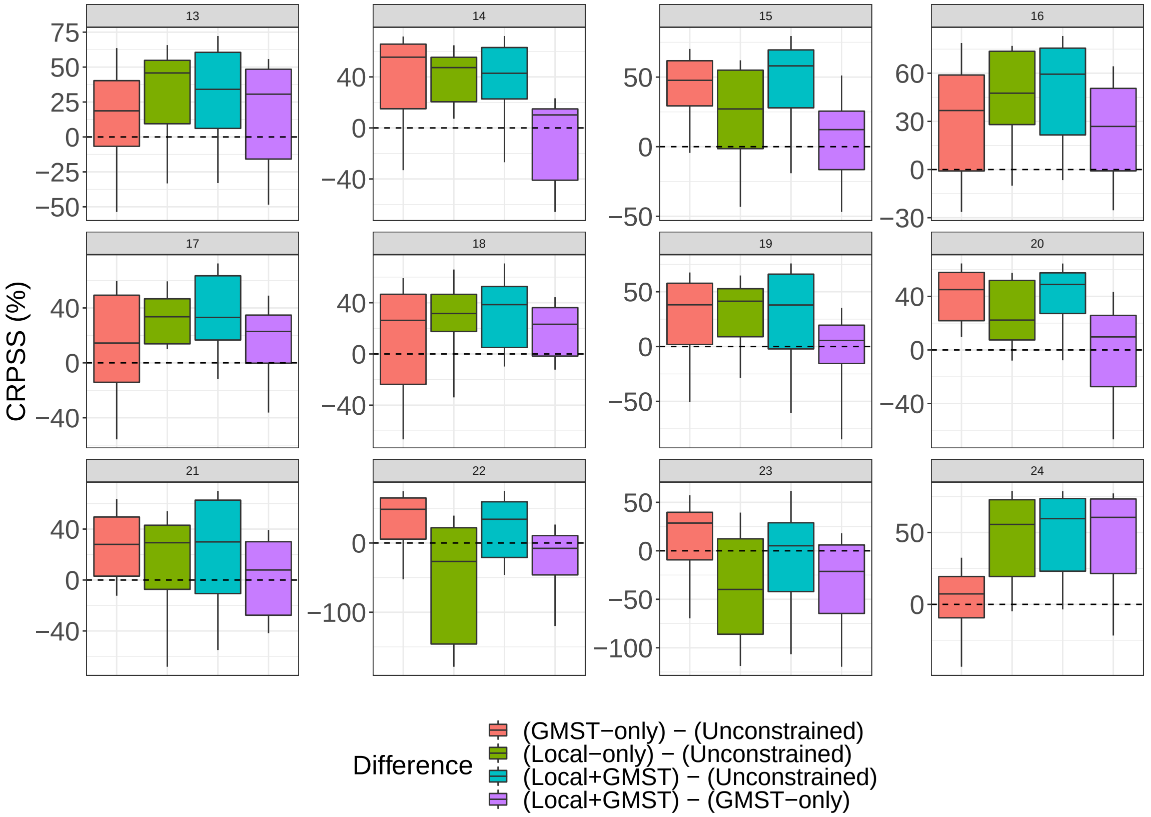

Fig. S 6: Same as Fig. S5 but for the points from 13 to 24.

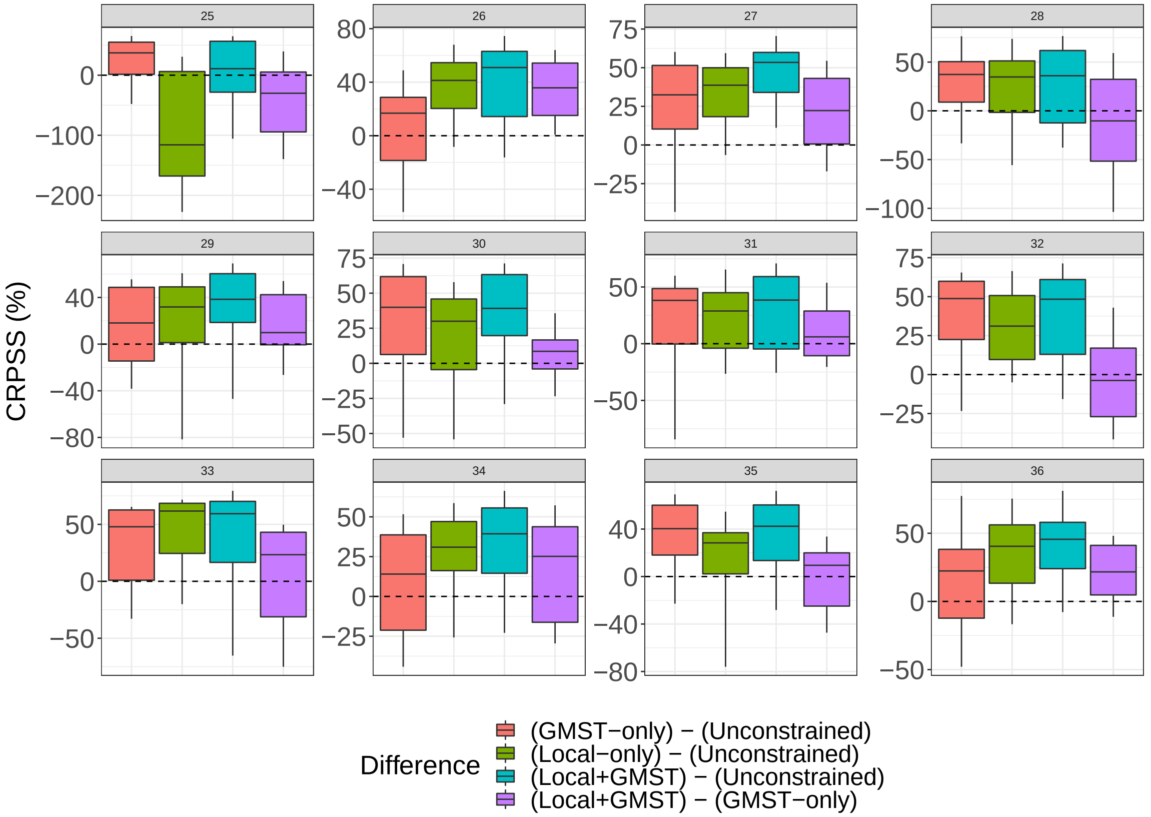

Fig. S 7: Same as Fig. S5 but for the points from 25 to 36.

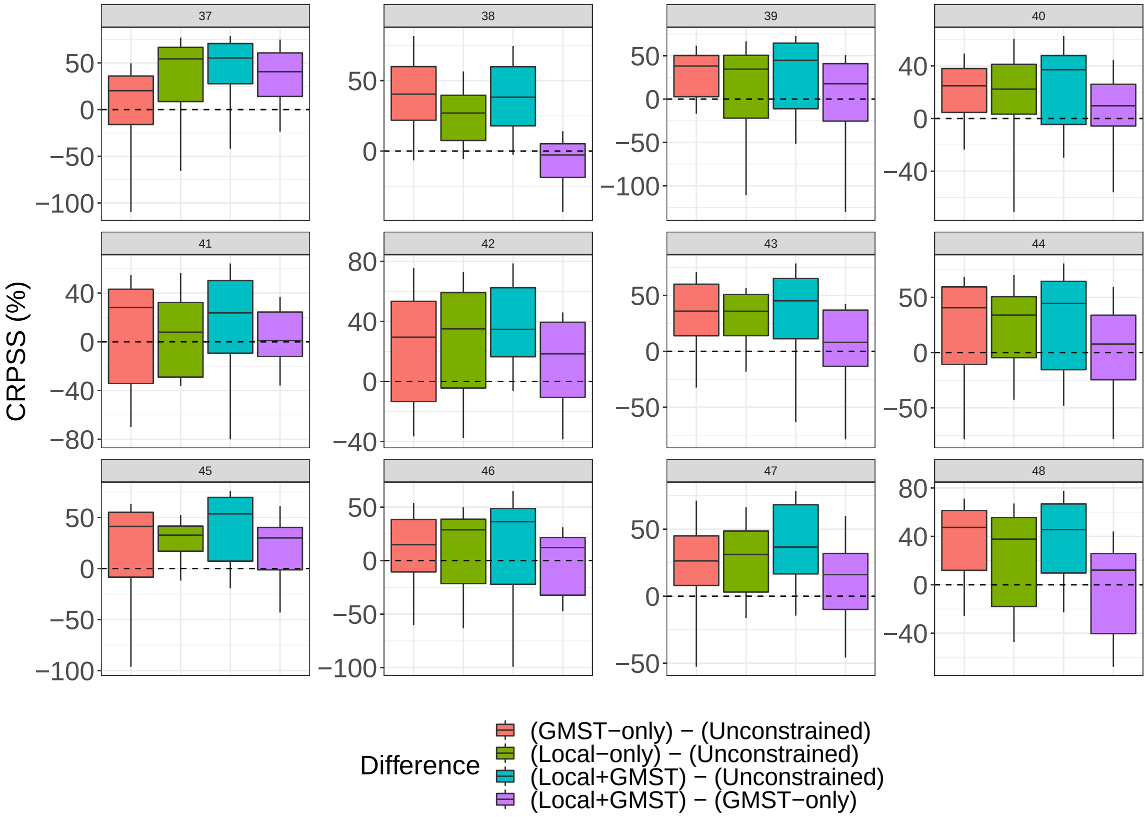

Fig. S 8: Same as Fig. S5 but for the points from 37 to 48.

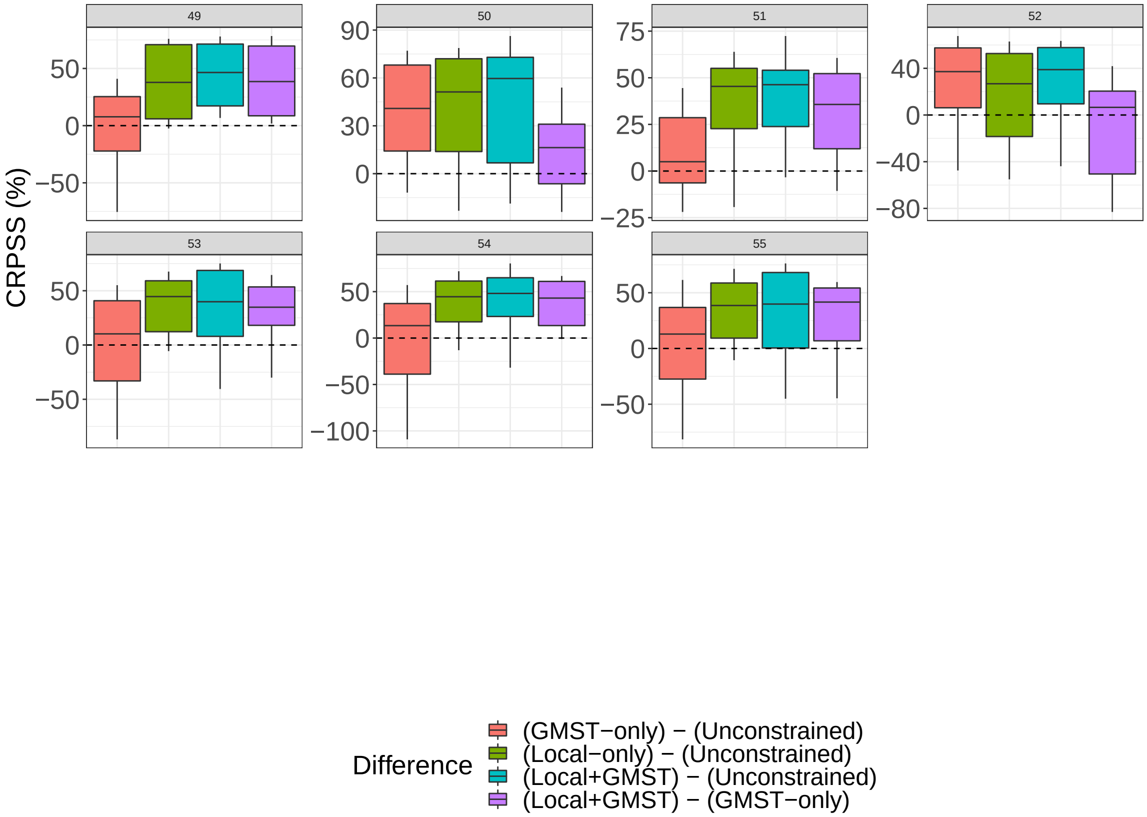

Fig. S 9: Same as Fig. S5 but for the points from 49 to 55.

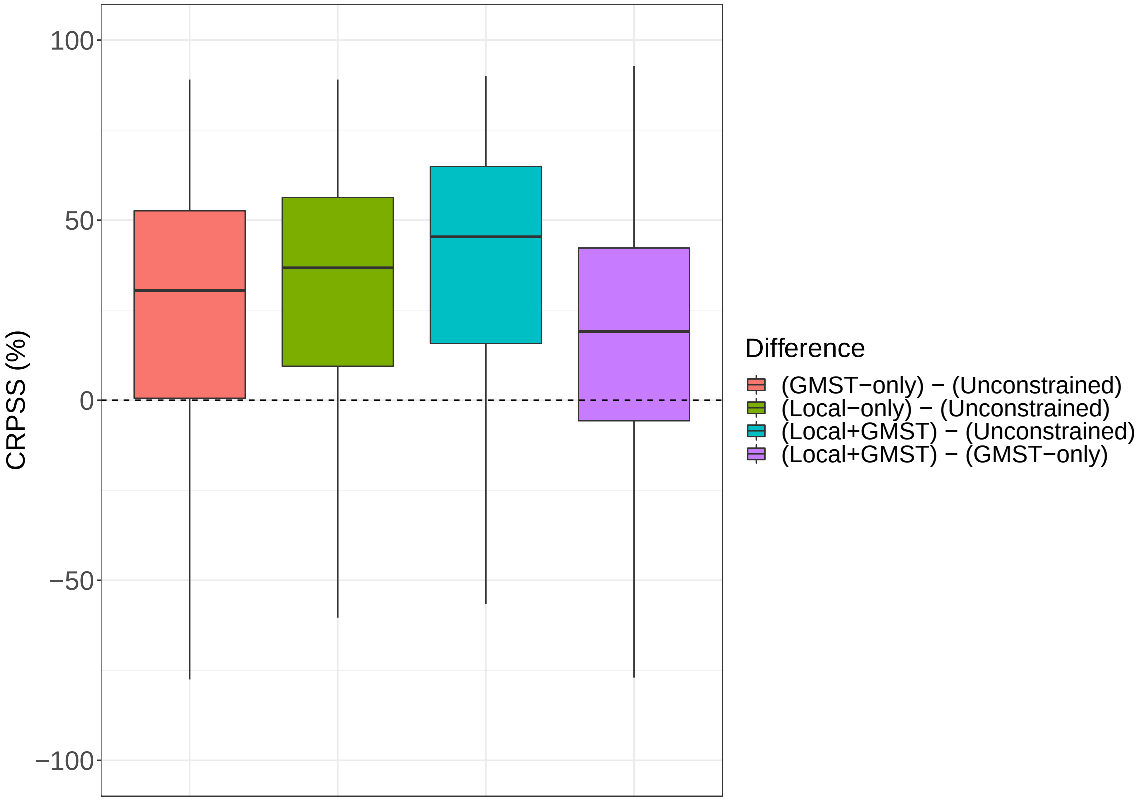

Fig. S 10: **CRPSS for the constrained temperature projections within the perfect model framework.** The red, green and blue boxplots indicate the CRPSS distributions of the GMST-only, Local-only and Local+GMST constraints, respectively, compared to the unconstrained case. Calculation is made for all locations (see points in Fig. 5) and models, over the 2021-2040 period. The boxplot in magenta indicates the added value of the local observations and stand for the CRPSS distributions in the Local+GMST case compared to the GMST-only case. The top (bottom) of the box represents the 25th (75th) percentile of the distribution and the upper (lower) whisker represents the 95th (5th) percentile. Values are normalised by the number of members in each model. A CRPSS of 0 (dashed line) indicates the absence of added value of the method.

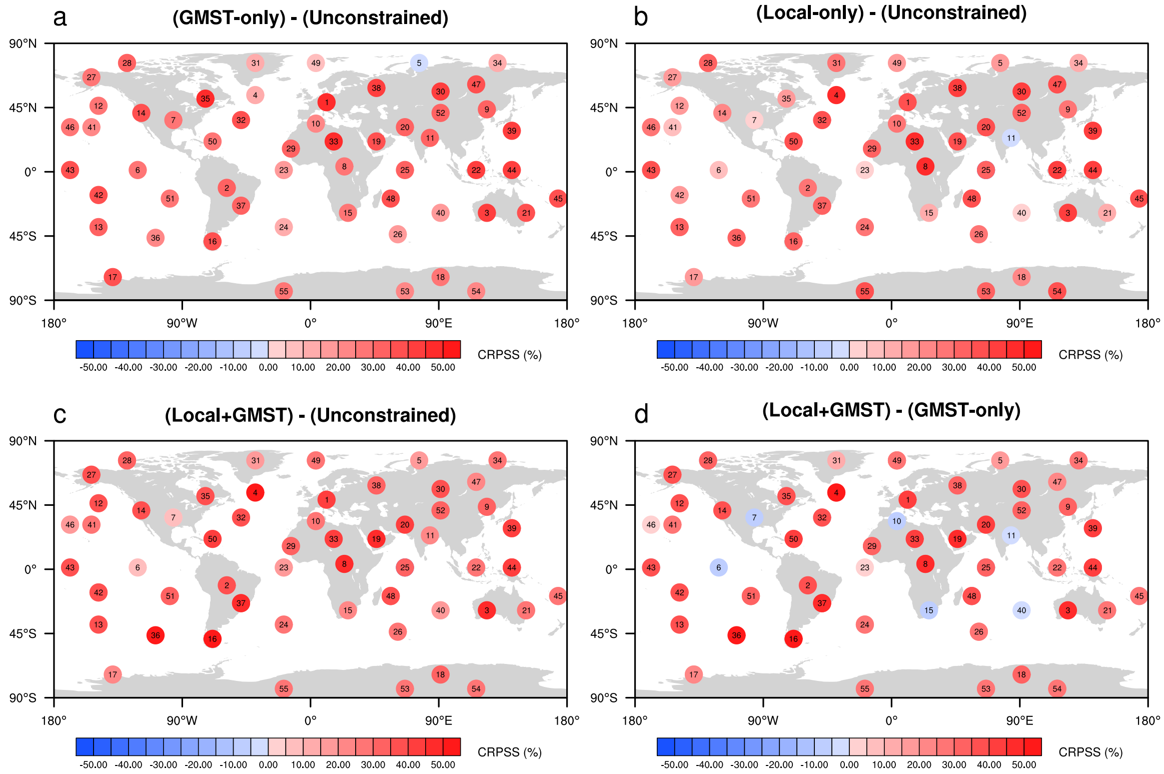

Fig. S 11: Same as Fig. 5 but for the 2081-2100 period.

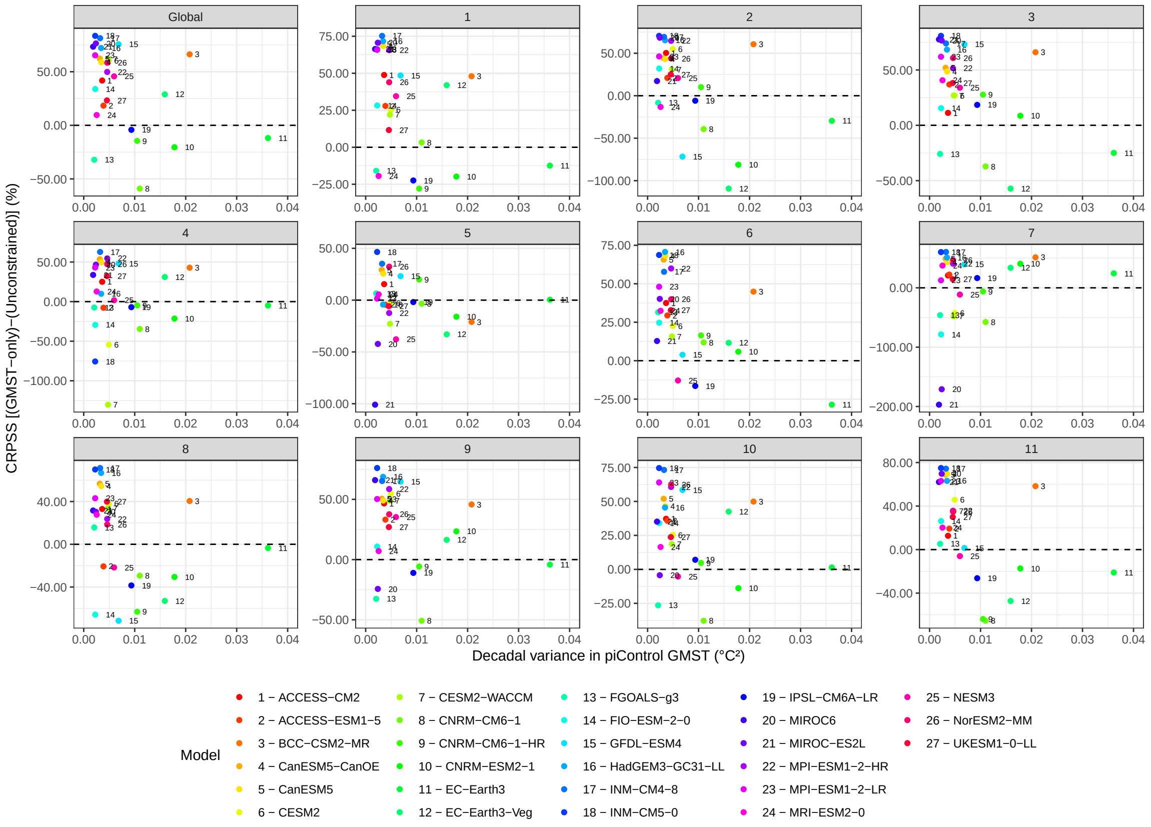

Fig. S 12: **Dependence of the skill of the method to the decadal variability.** CRPSS (in the GMST-only case compared to the unconstrained case) averaged over all available members (or pseudo-observations) from the same model (one point per model) for each location (see points in Fig. 5) vs the decadal variance in the associated low-pass filtered GMST from the pre-industrial control (piControl) simulation.

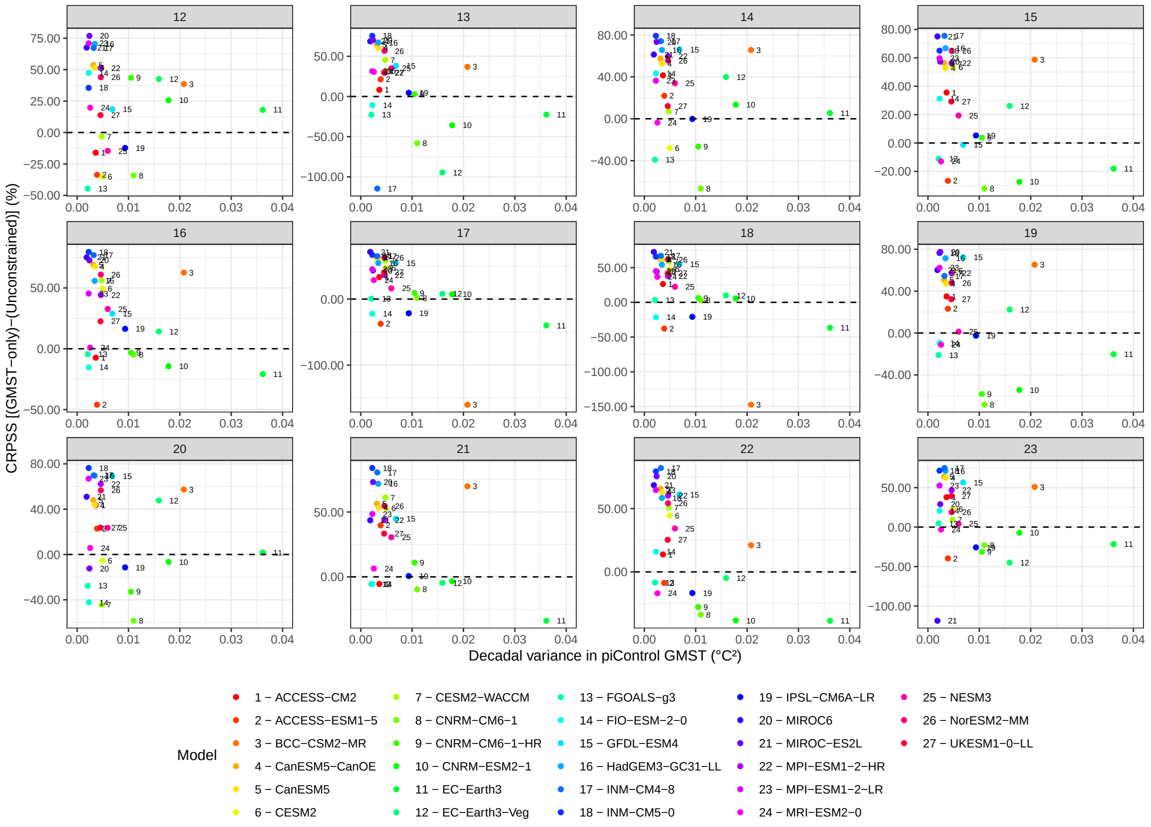

Fig. S 13: Same as Fig. S12 but for the points from 12 to 23.

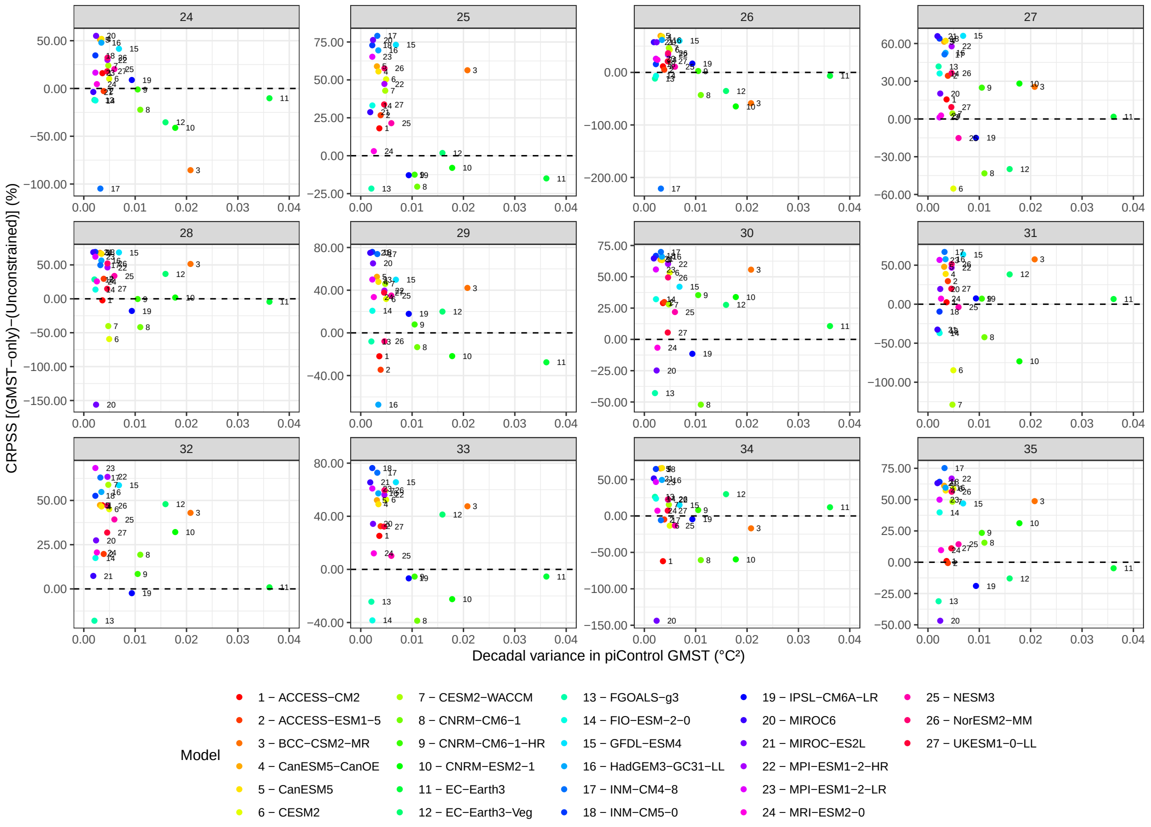

Fig. S 14: Same as Fig. S12 but for the points from 24 to 35.

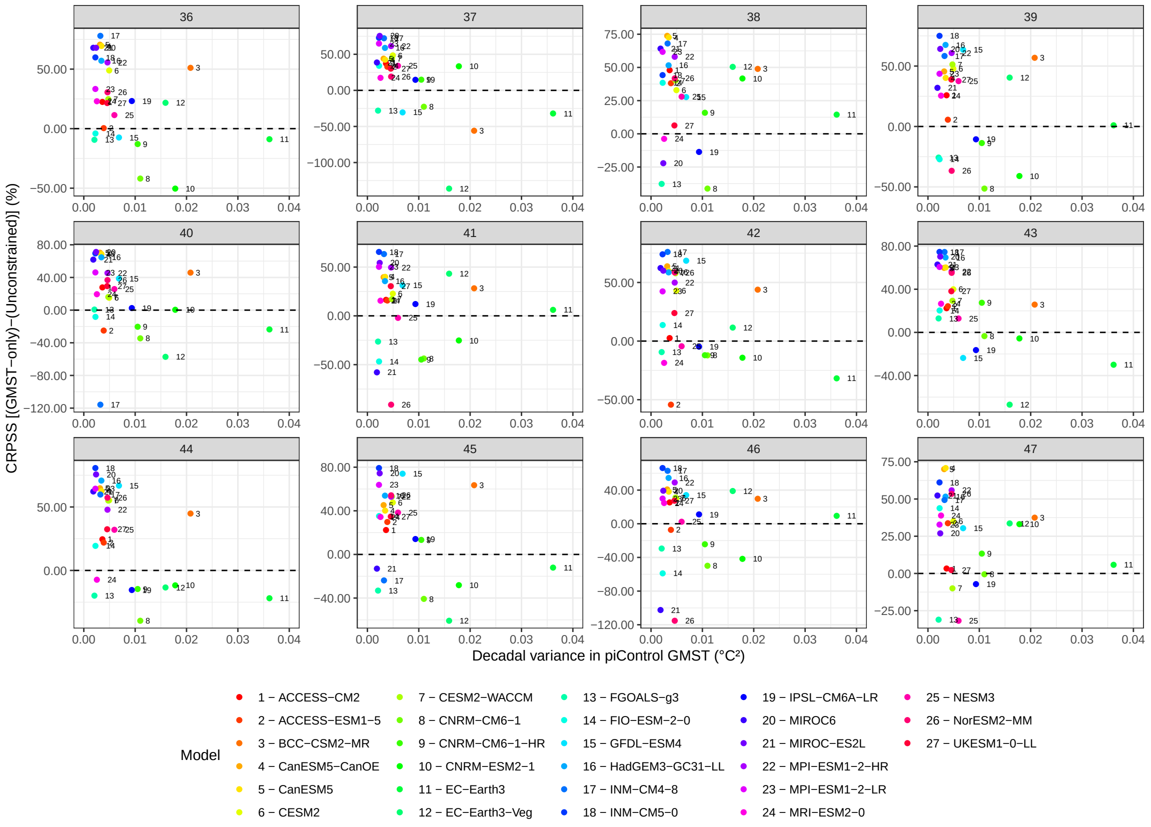

Fig. S 15: Same as Fig. S12 but for the points from 36 to 47.

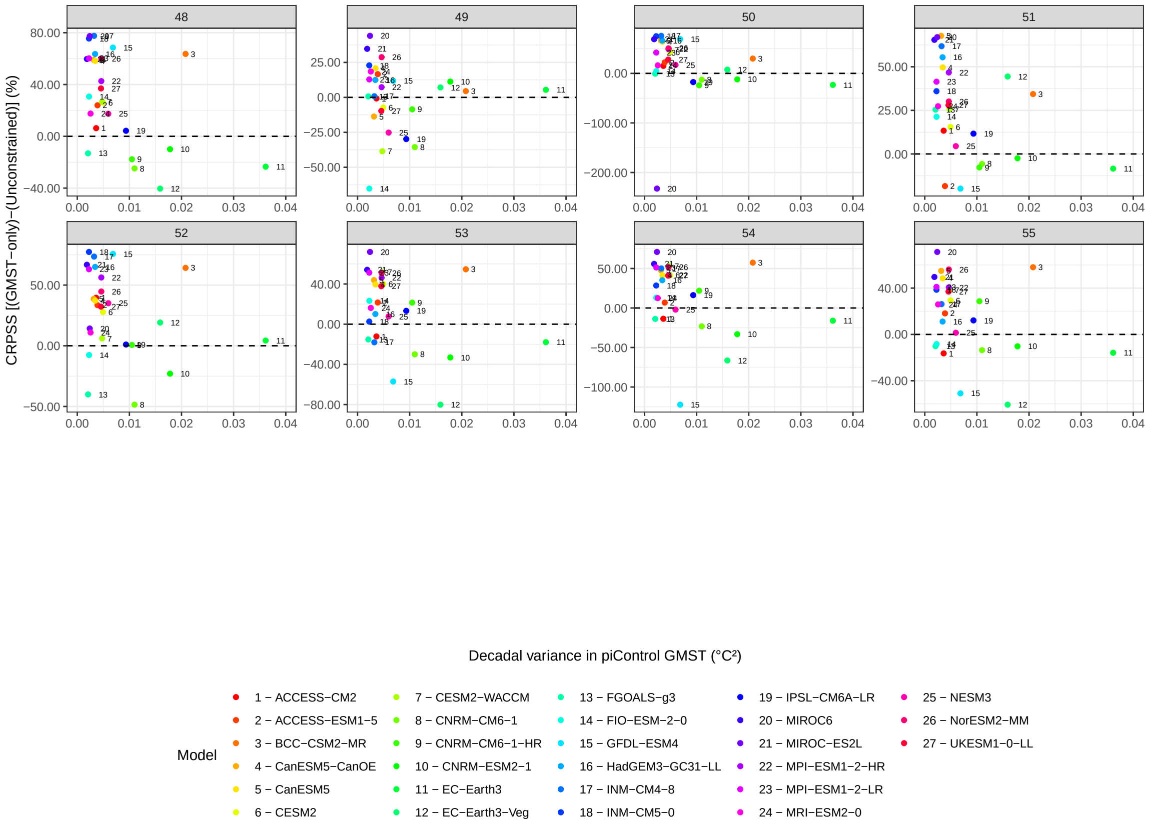

Fig. S 16: Same as Fig. S12 but for the points from 48 to 55.

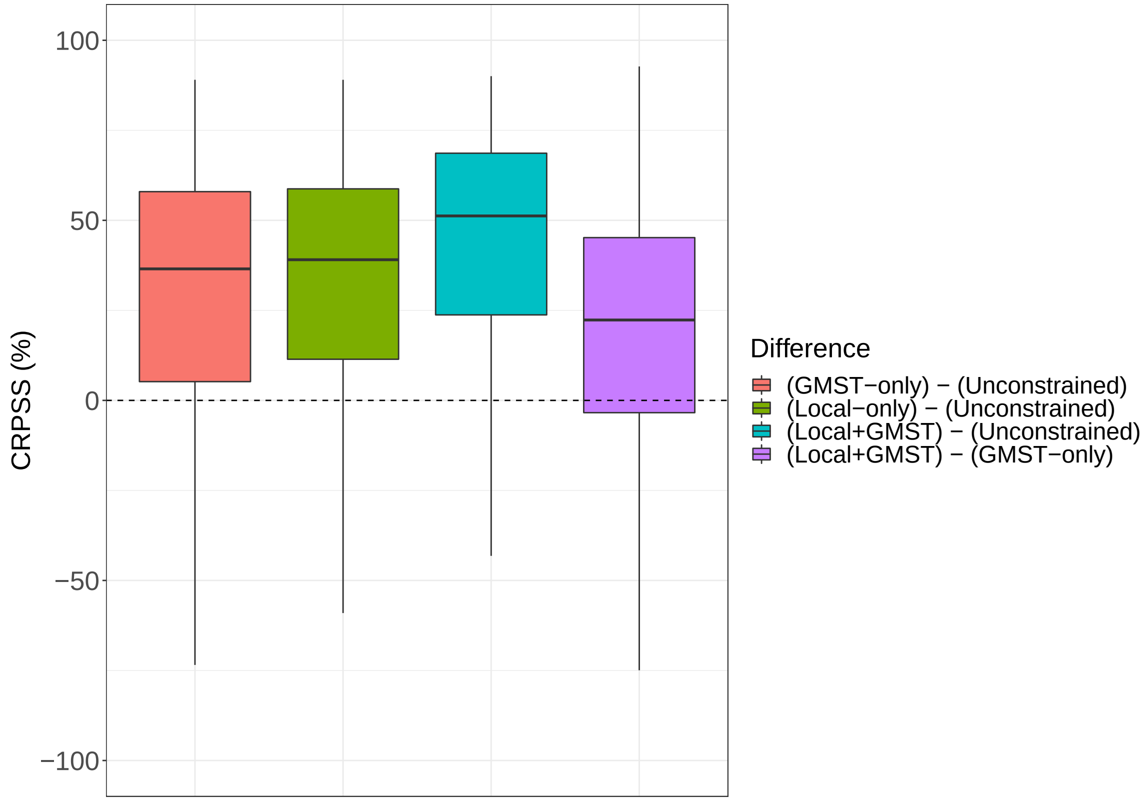

Fig. S 17: **CRPSS for the constrained temperature projections over the 2021-2040 period within the perfect model framework.** Same as Fig. S10 but without the models characterised by a strong decadal internal variability (CNRM-CM6-1, CNRM-CM6-1-HR, CNRM-ESM2-1, EC-Earth3, EC-Earth3-Veg, IPSL-CM6A-LR, BCC-CSM2-MR).

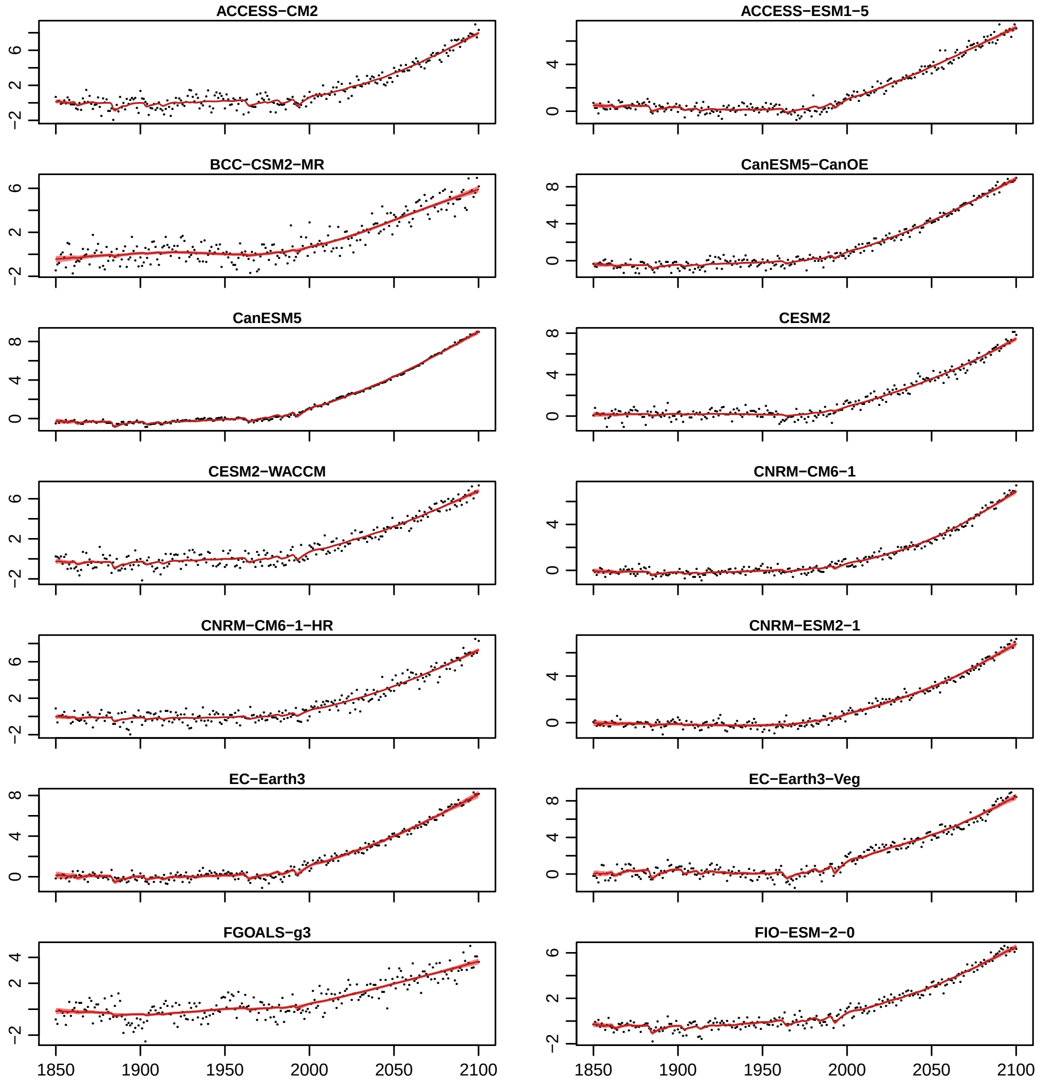

Fig. S 18: **Filtering of historical + SSP5-8.5 simulations.** For each CMIP6 model, we estimate the total forced response in historical and SSP5-8.5 simulations for a given grid point over Europe (number 1 in Fig. 5, following the procedure described in the Methods section. Shown are the best estimate (solid line) and 5-95% range (shading; these ranges are quite narrow and, as a consequence, hardly visible).

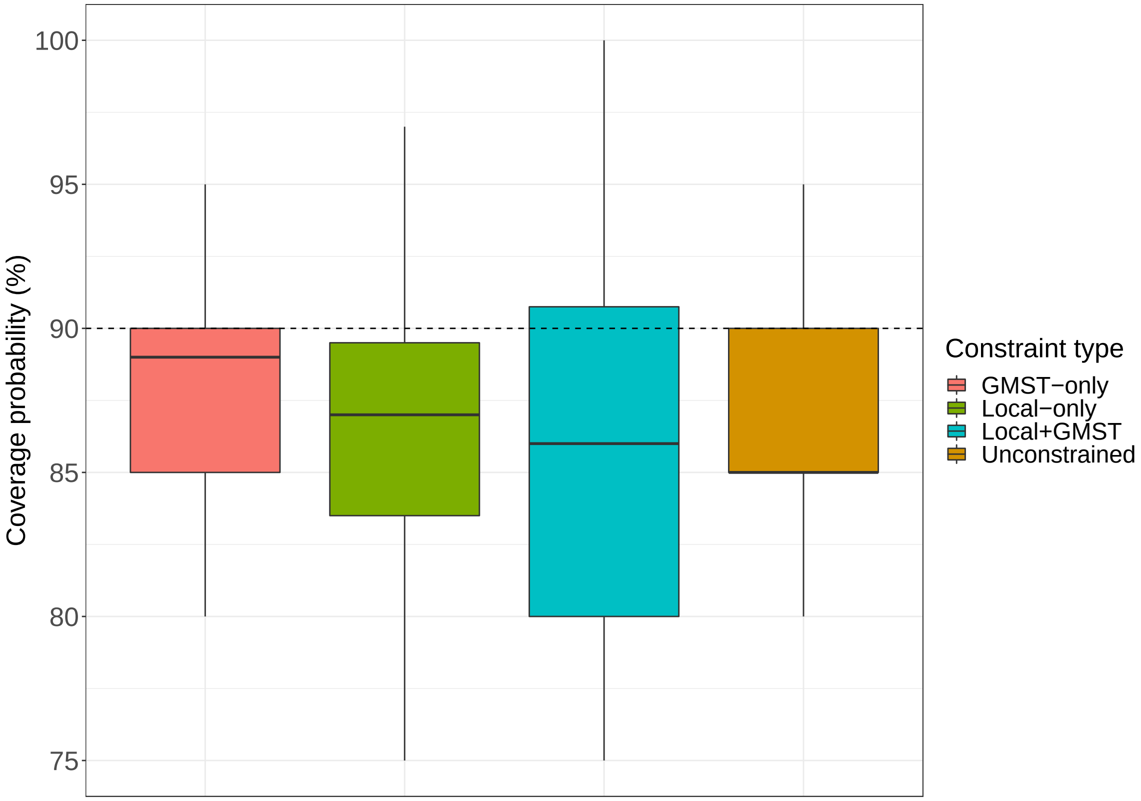

Fig. S 19: **Coverage probabilities of the constraining method applied for the local temperature over the 2021-2040 period within a perfect model framework.** Same as Fig. S4 but without the models characterised by a strong decadal internal variability (CNRM-CM6-1, CNRM-CM6-1-HR, CNRM-ESM2-1, EC-Earth3, EC-Earth3-Veg, IPSL-CM6A-LR, BCC-CSM2-MR).

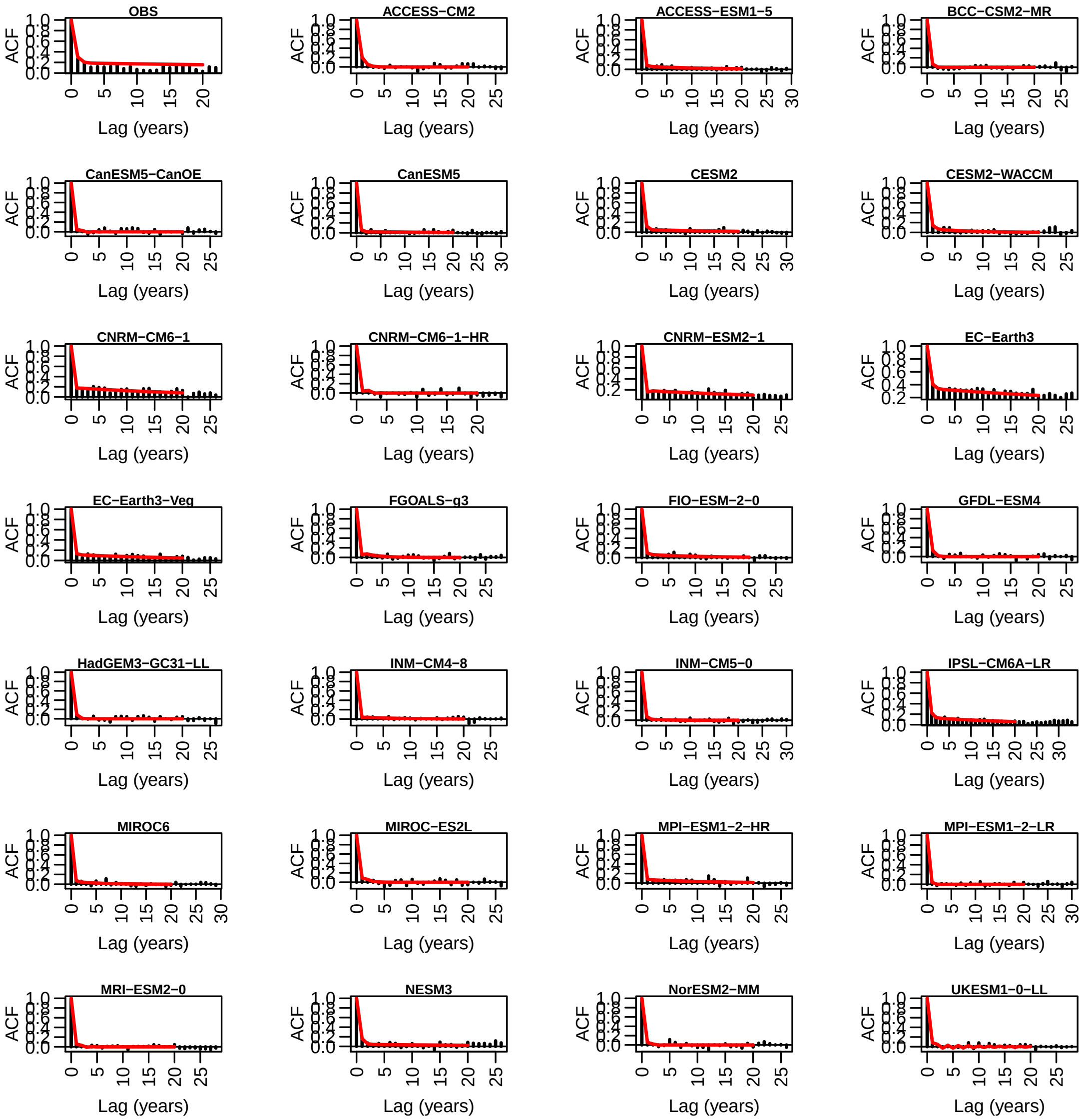

Fig. S 20: **Internal variability in CMIP6 piControl simulations compared to the observed estimate.** For each model, the auto-correlation function (bars) of the piControl temperature time series for the point over Europe (number 1) is to be compared to the MAR process fitted on the observed residuals (first panel).

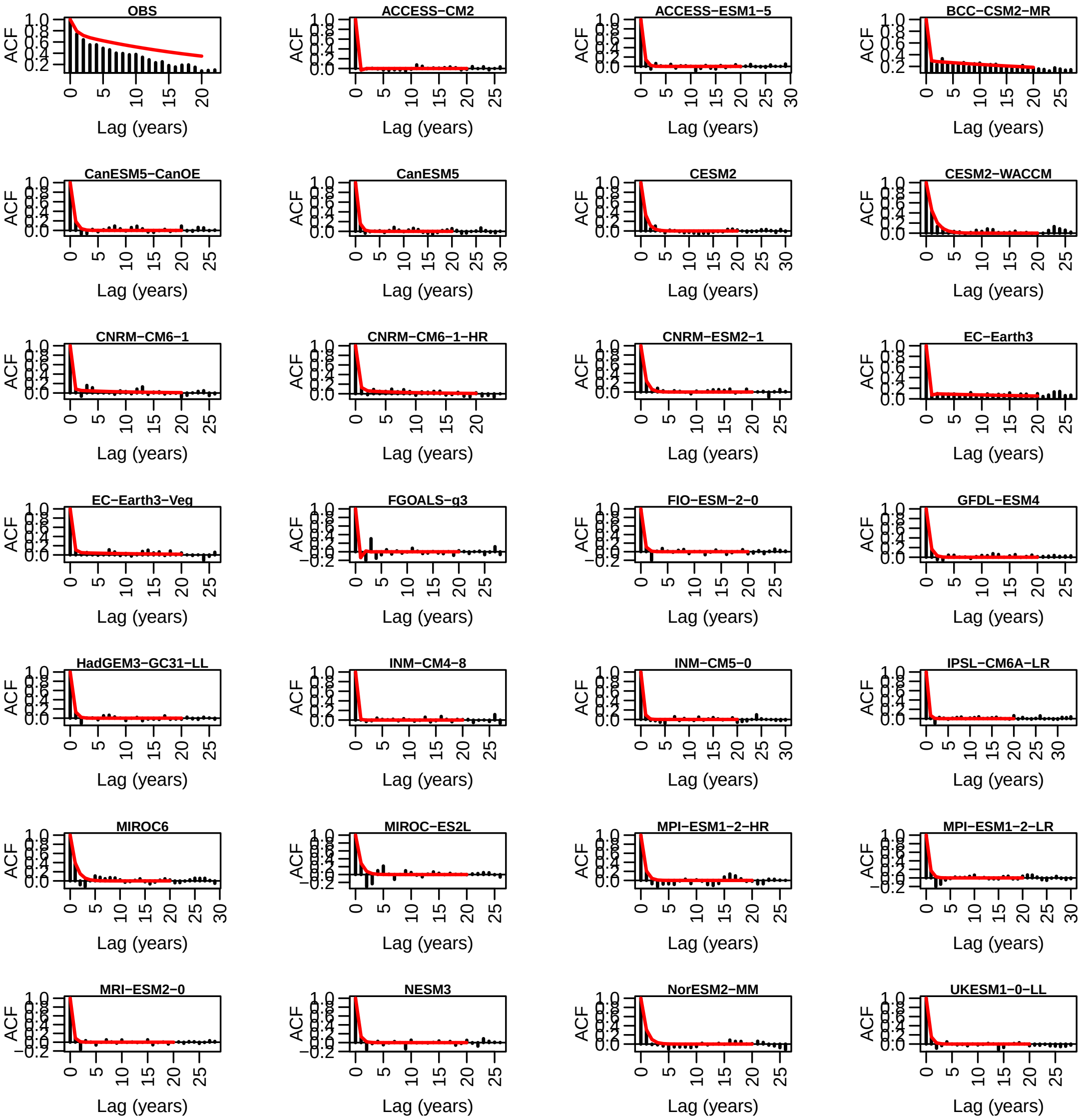

Fig. S 21: Same as Fig. S20 but for the point over South America (number 2).

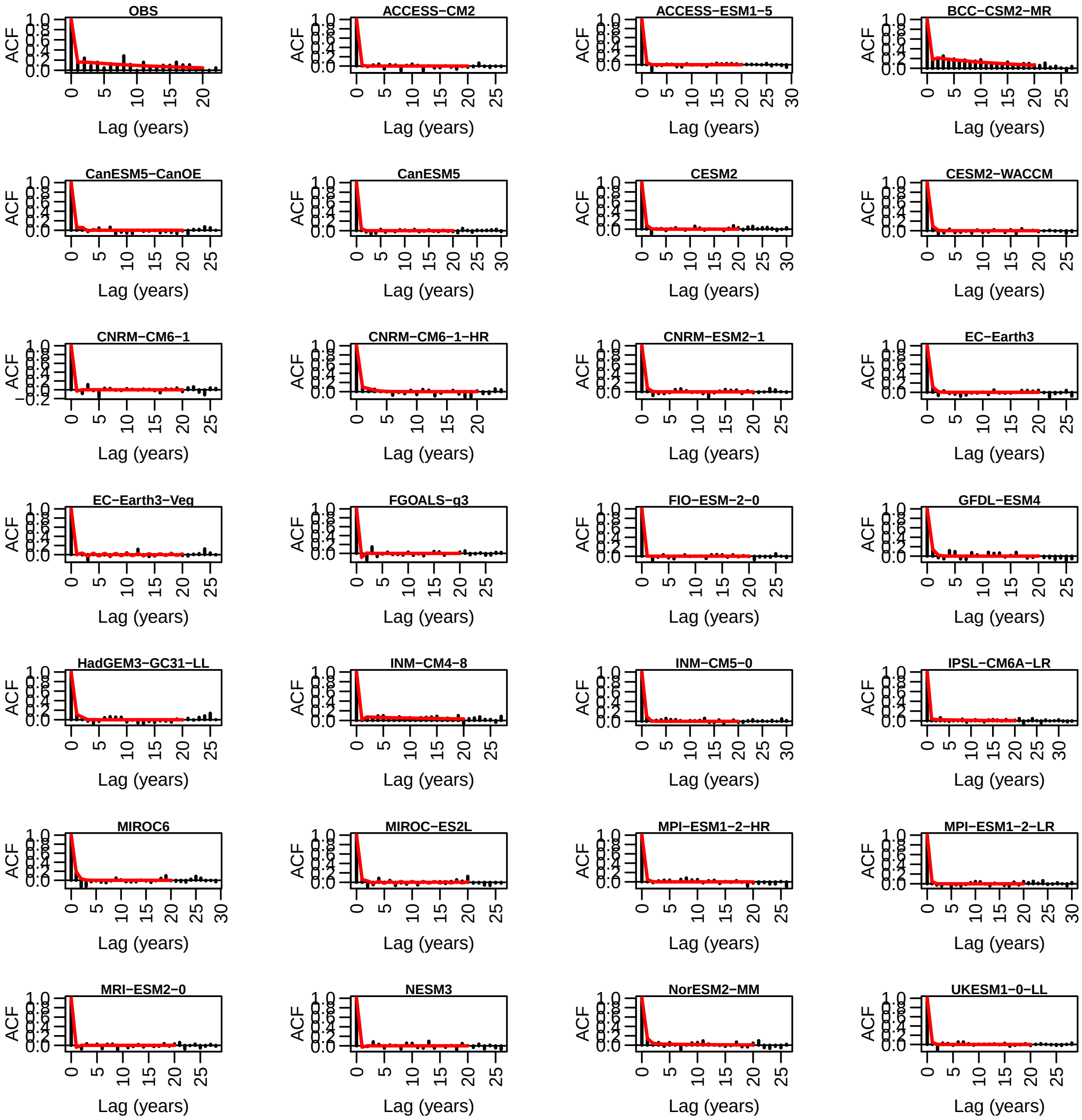

Fig. S 22: Same as Fig. S20 but for the point over Australia (number 3).

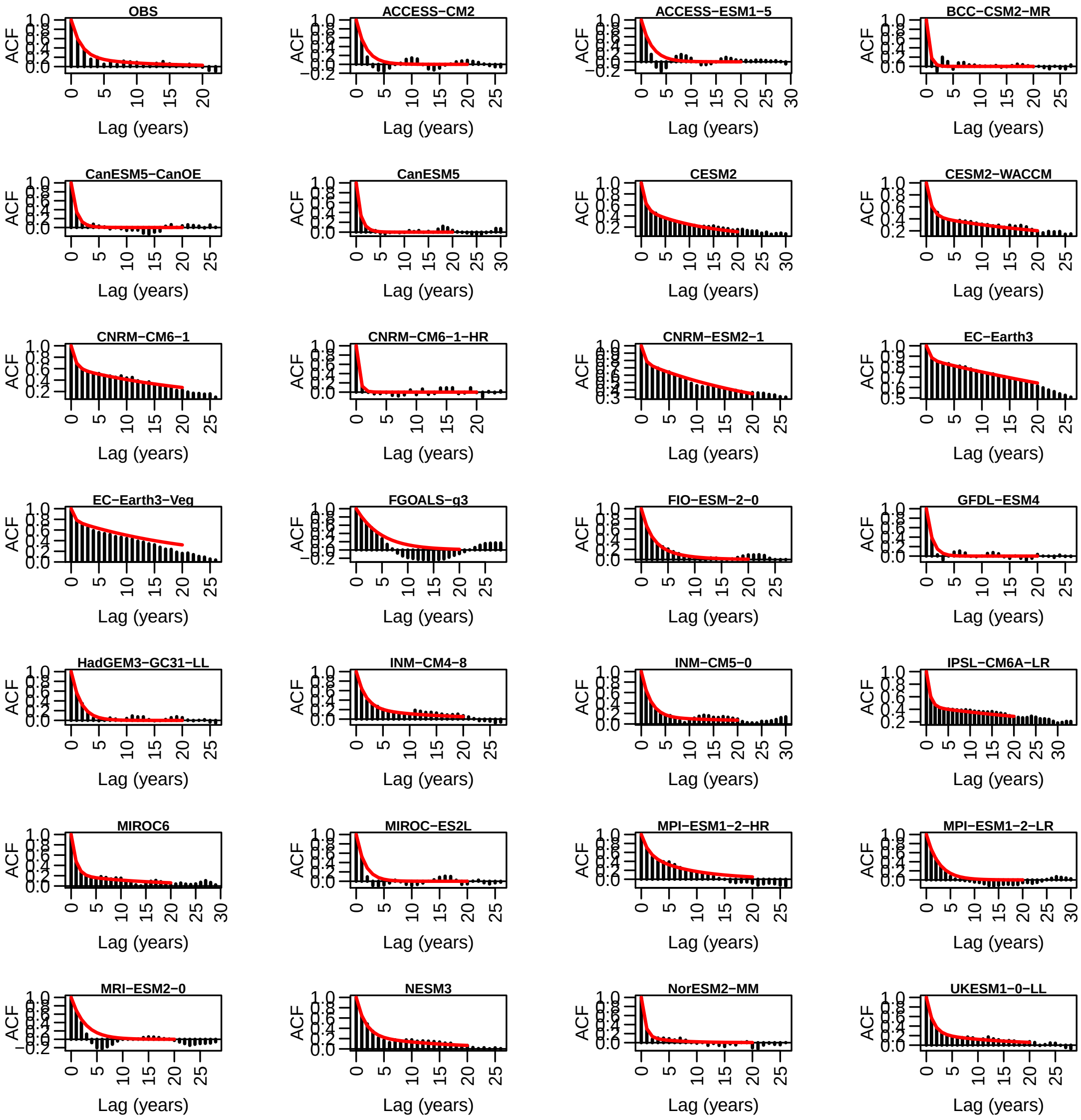

Fig. S 23: Same as Fig. S20 but for the point over the Atlantic ocean (number 4).

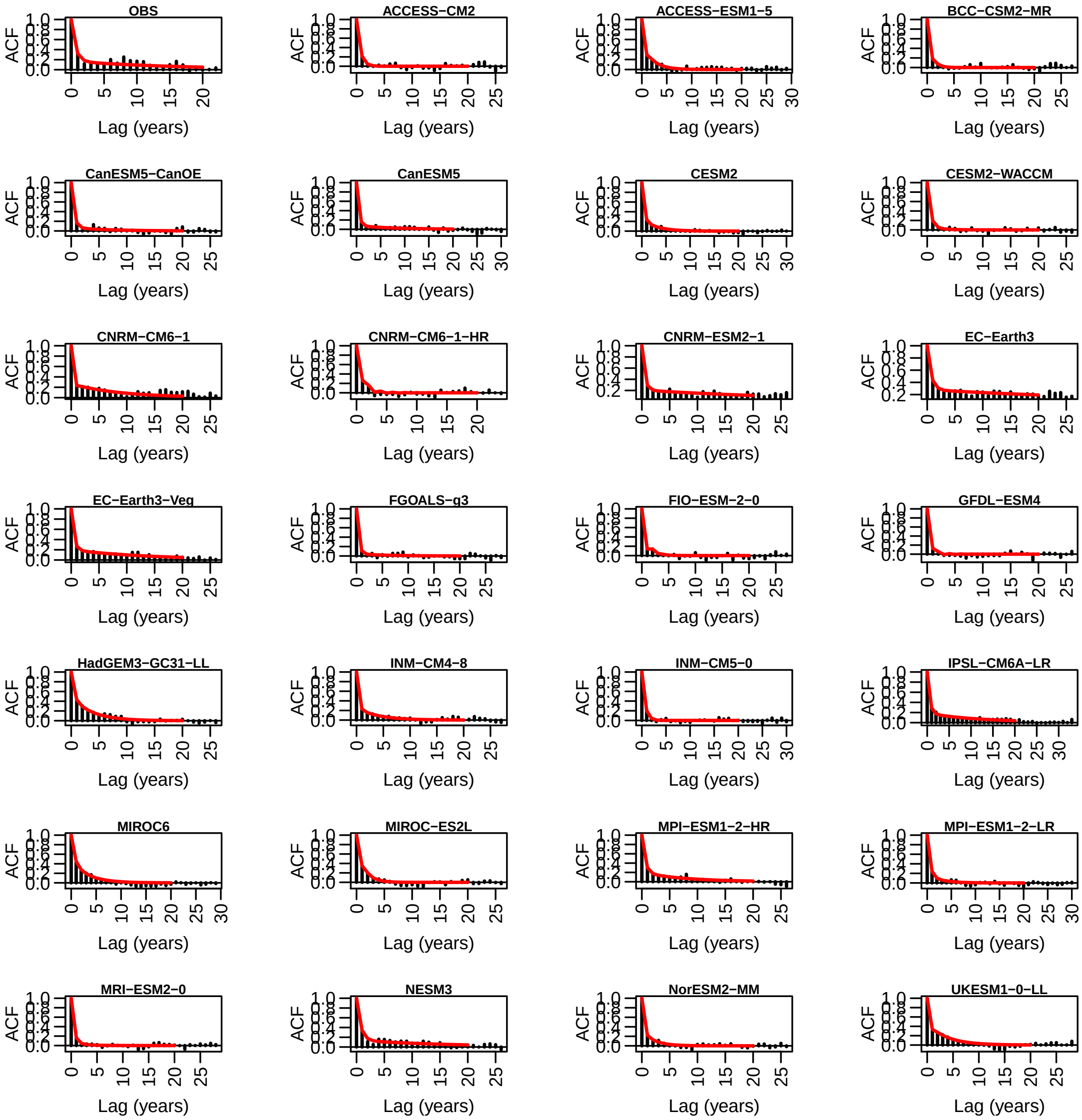

Fig. S 24: Same as Fig. S20 but for the point over the Arctic (number 5).

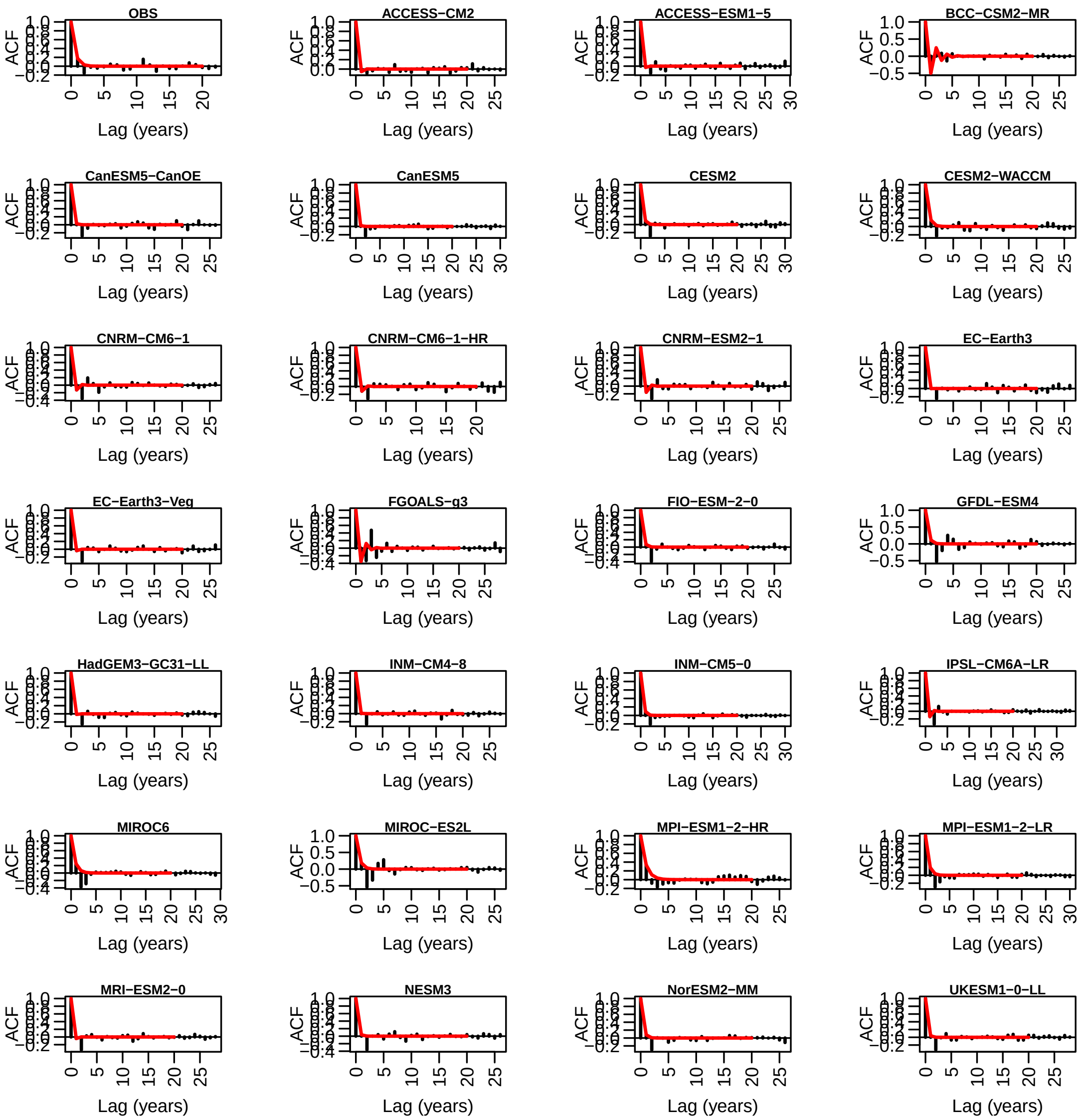

Fig. S 25: Same as Fig. S20 but for the point over the Pacific ocean (number 6).

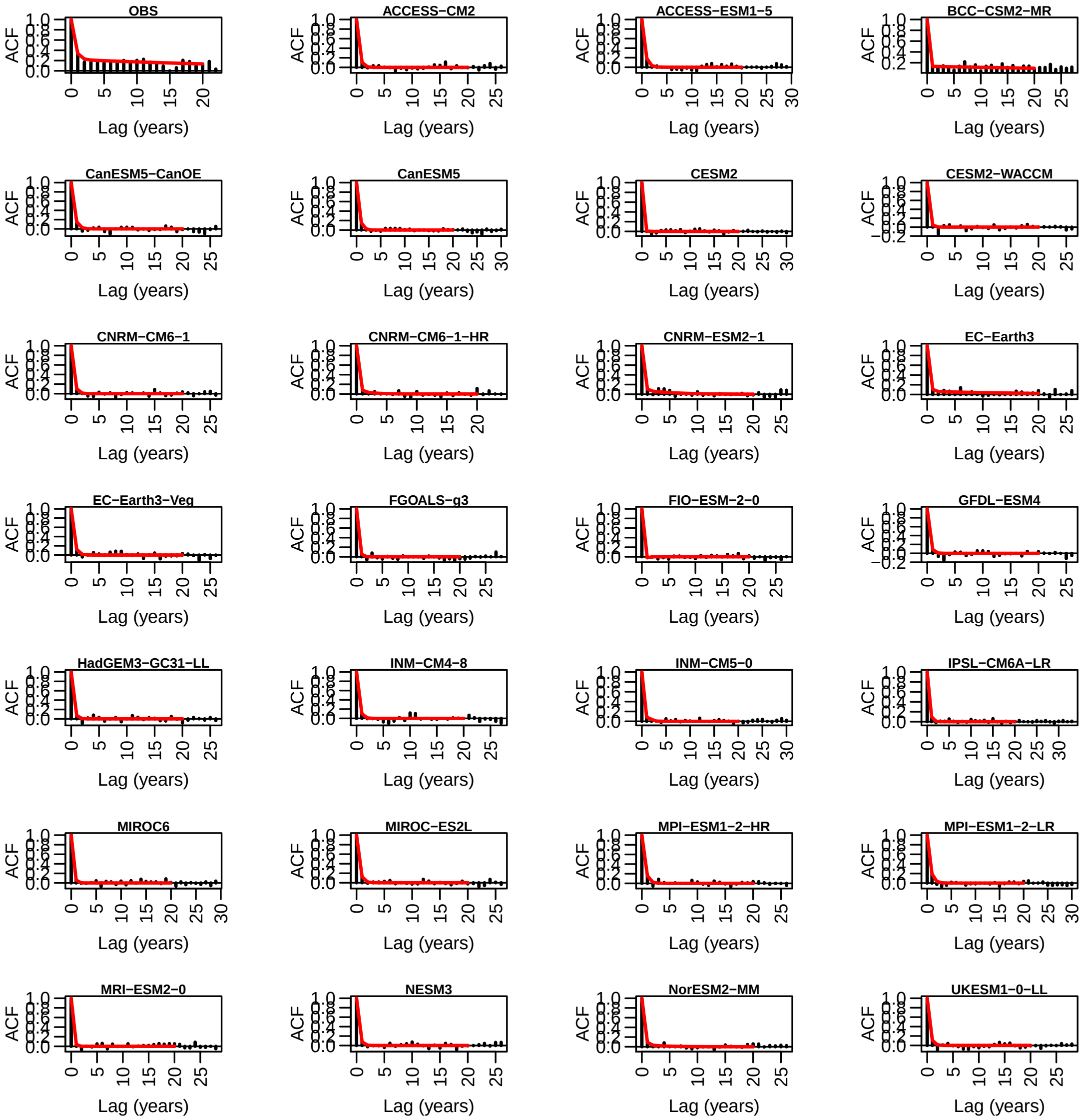

Fig. S 26: Same as Fig. S20 but for the point over North America (number 7).

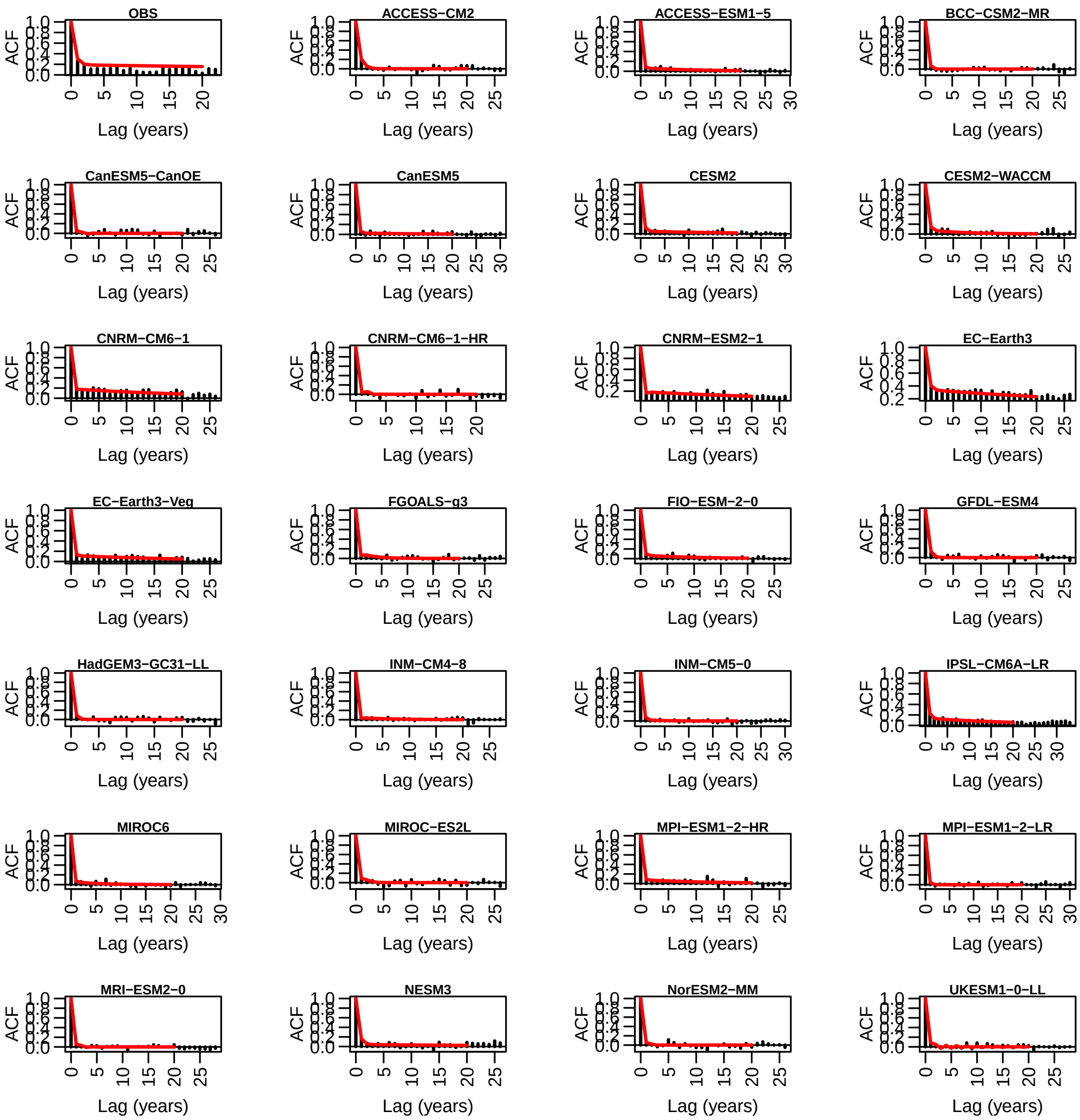

Fig. S 27: Same as Fig. S20 but for the point over Africa (number 8).

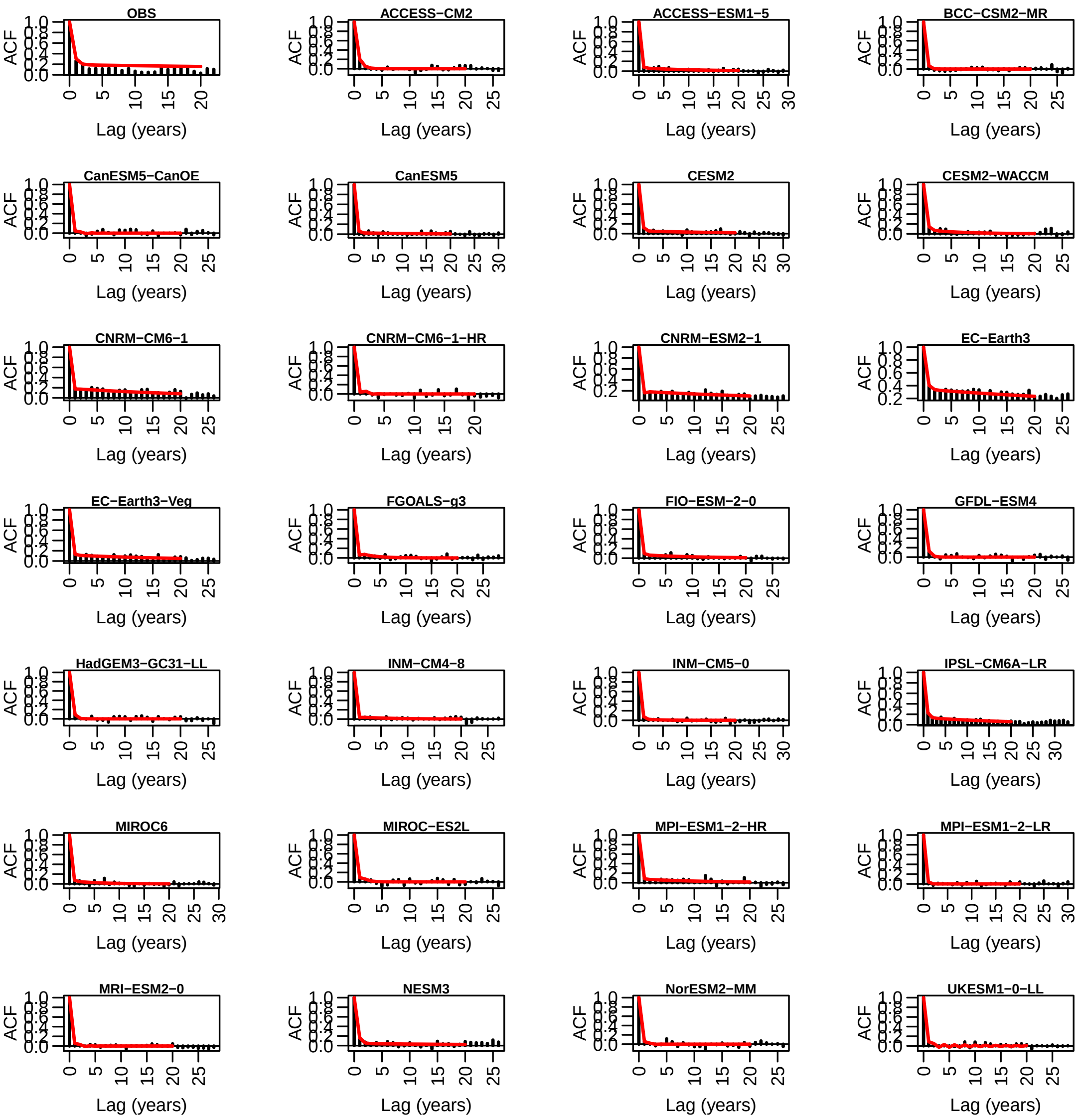

Fig. S 28: Same as Fig. S20 but for the point over East Asia (number 9).

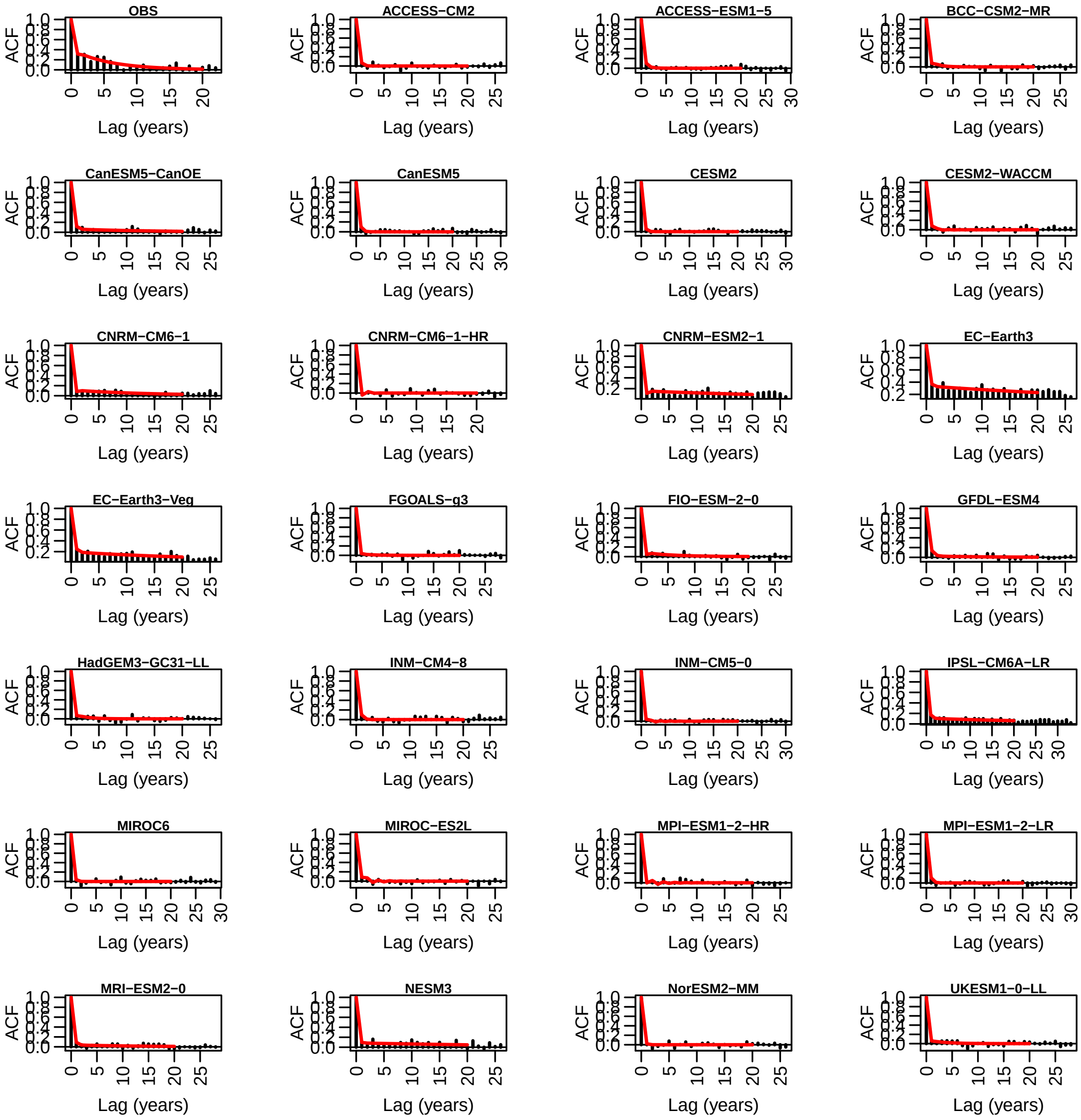

Fig. S 29: Same as Fig. S20 but for the point over the Mediterranean (number 10).

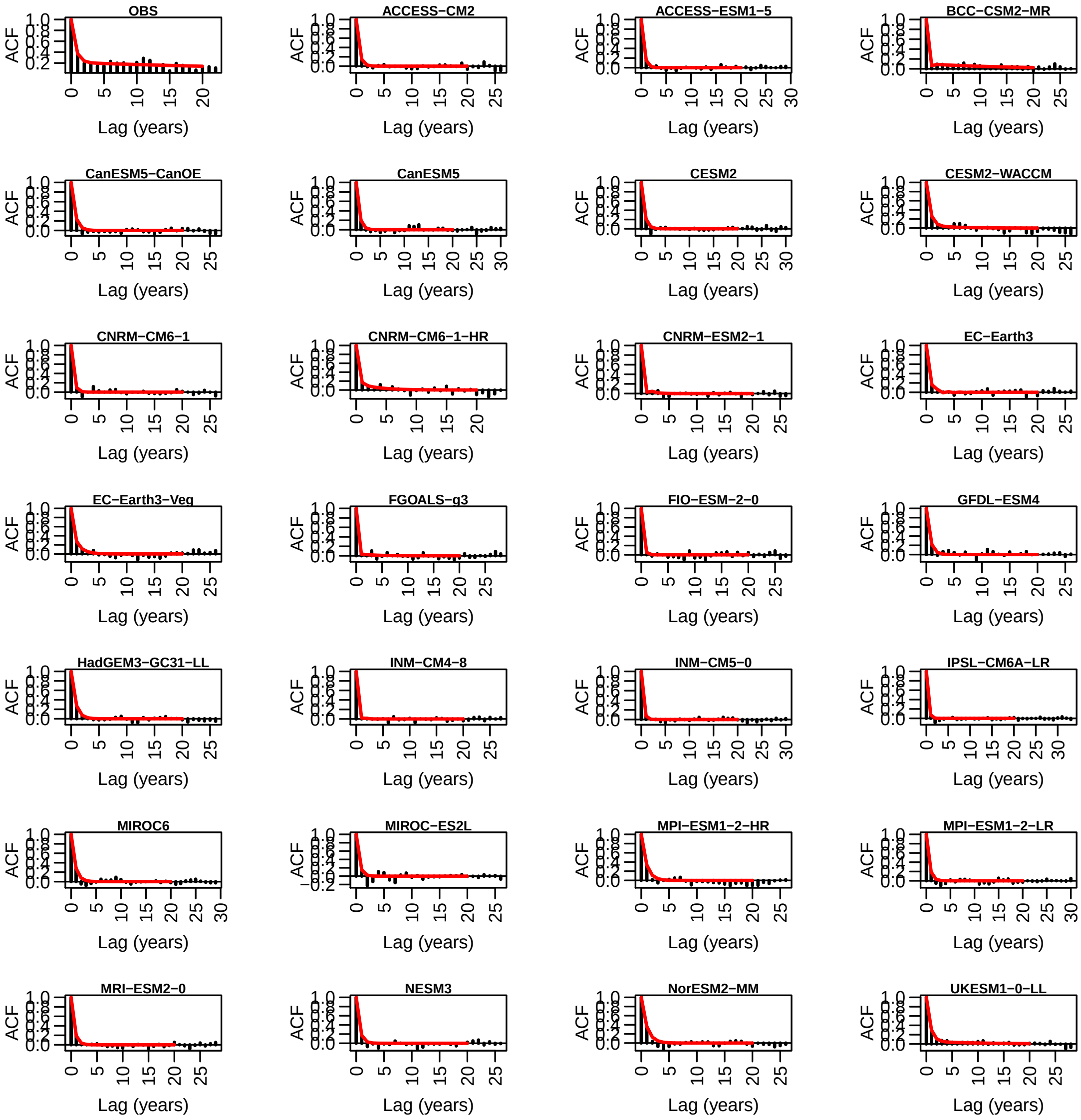

Fig. S 30: Same as Fig. S20 but for the point over South Asia (number 11).

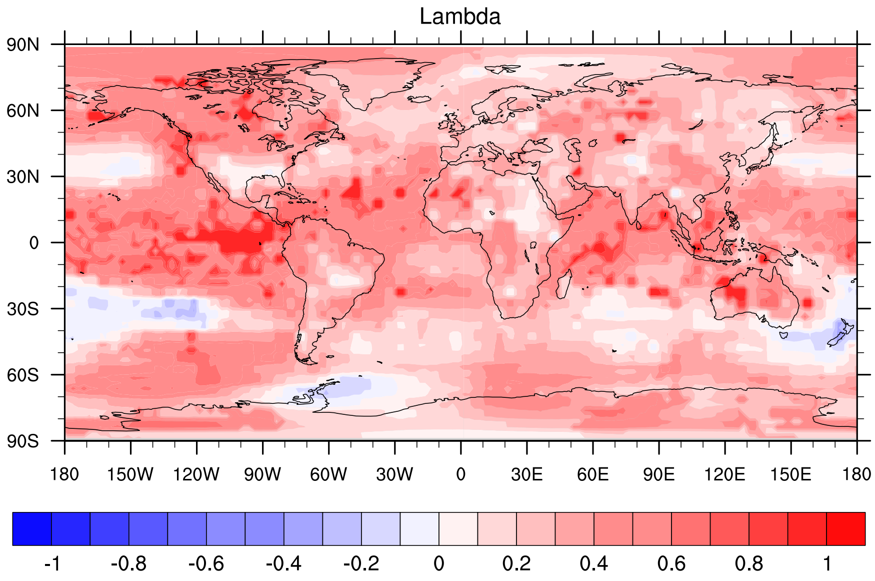

Fig. S 31: **Dependency between the GMST and local temperature residuals.** Spatial pattern of the parameter λ, modeling the dependence between GMST and local temperature residuals.
